# Neutralizing Carbapenem Resistance by Co-Administering Meropenem with Novel β-Lactam-Metallo-β-Lactamase Inhibitors

**DOI:** 10.3390/antibiotics12040633

**Published:** 2023-03-23

**Authors:** Nakita Reddy, Letisha Girdhari, Mbongeni Shungube, Arnoldus C. Gouws, Byron K. Peters, Kamal K. Rajbongshi, Sooraj Baijnath, Sipho Mdanda, Thandokuhle Ntombela, Thilona Arumugam, Linda A. Bester, Sanil D. Singh, Anil Chuturgoon, Per I. Arvidsson, Glenn E. M Maguire, Hendrik G. Kruger, Thavendran Govender, Tricia Naicker

**Affiliations:** 1 Catalysis and Peptide Research Unit, University of KwaZulu-Natal, Durban 4001, South Africa; nakita236@gmail.com (N.R.); 216001542@stu.ukzn.ac.za (L.G.);; 2 School of Physiology, Faculty of Health Sciences, University of the Witwatersrand, Johannesburg 2020, South Africa; 3 School of Laboratory Medicine and Medical Sciences, College of Health Sciences, University of KwaZulu-Natal, Durban 4000, South Africa; 4 Department of Pharmaceutical Sciences, University of KwaZulu-Natal, Westville Campus, Durban 3629, South Africa; 5 Science for Life Laboratory, Drug Discovery & Development Platform & Division of Translational Medicine and Chemical Biology, Department of Medical Biochemistry and Biophysics, Karolinska Institutet, 17177 Stockholm, Sweden; 6 School of Chemistry and Physics, University of KwaZulu-Natal, Durban 4001, South Africa; 7 Department of Chemistry, University of Zululand, Private Bag X1001, KwaDlangezwa 3886, South Africa

**Keywords:** metallo-β-lactamases, Carbapenem resistant *Enterobacterales*, cyclic amino acidic chelator, BP2, murine thigh infection model

## Abstract

Virulent Enterobacterale strains expressing serine and metallo-β-lactamases (MBL) genes have emerged responsible for conferring resistance to hard-to-treat infectious diseases. One strategy that exists is to develop β-lactamase inhibitors to counter this resistance. Currently, serine β-lactamase inhibitors (SBLIs) are in therapeutic use. However, an urgent global need for clinical metallo-β-lactamase inhibitors (MBLIs) has become dire. To address this problem, this study evaluated BP2, a novel beta-lactam-derived β-lactamase inhibitor, co-administered with meropenem. According to the antimicrobial susceptibility results, BP2 potentiates the synergistic activity of meropenem to a minimum inhibitory concentration (MIC) of ≤1 mg/L. In addition, BP2 is bactericidal over 24 h and safe to administer at the selected concentrations. Enzyme inhibition kinetics showed that BP2 had an apparent inhibitory constant (K*_i_*_app_) of 35.3 µM and 30.9 µM against New Delhi Metallo-β-lactamase (NDM-1) and Verona Integron-encoded Metallo-β-lactamase (VIM-2), respectively. BP2 did not interact with glyoxylase II enzyme up to 500 µM, indicating specific (MBL) binding. In a murine infection model, BP2 co-administered with meropenem was efficacious, observed by the >3 log_10_ reduction in *K. pneumoniae* NDM cfu/thigh. Given the promising pre-clinical results, BP2 is a suitable candidate for further research and development as an (MBLI).

## 1. Introduction

The emergence of the global COVID-19 pandemic has exacerbated the antibiotic crisis. Many COVID-19 patients fell victim to secondary Enterobacterale infections, in which the symptoms could not be differentiated from those of COVID-19, resulting in the demise of an already crippled healthcare system [1]. The number of circulating Carbapenem-Resistant Enterobacterales (CRE) strains has thus tremendously increased in recent years [2,3]. Since β-lactams are the most widely used class of antibiotics [4,5], this poses a massive challenge to clinicians, with carbapenems being considered the “last line of defense in the treatment of infectious disease” [6]. CREs producing β-lactamases are classified as either SBLs or MBLs [7,8]. Currently, the most difficult-to-treat infections emanate from CREs producing MBL enzymes [4,9]. These enzymes hydrolyze the β-lactam ring of the carbapenem antibiotic via the utilization of zinc ions at the active site, which creates a nucleophilic molecule, thereby rendering the β-lactam antibiotic ineffective [10,11].

The most prevalent MBLs globally are variants of NDM, VIM, and Imipenemase (IMP) [12,13,14,15]. There has been successful progress in developing several FDA-approved SBLIs for clinical use [16,17,18,19,20]. However, progress towards an efficacious MBLI has been stagnant; although research is ongoing [21], there has only been a handful of MBLI reaching clinical trials [22], e.g., the recent dual boronic acid prodrug, QPX7728 [23]. Potential MBLIs to enter clinical trials should be novel, possess a good antimicrobial susceptibility profile, be safe and non-toxic, and show activity over a considerable amount of time [24,25].

Metal chelators have demonstrated activity against MBLs by sequestration of Zn^2+^ ions in the enzyme’s active site, thus rendering the lactamases inactive against the β-lactam. The CRE’s are re-sensitized through this process to the β-lactam antibiotic drug [26]. The use of metal chelators as a mechanism of combating MBLs dates to the early twenty-first century when metal chelators were found to inhibit the MBL VIM-2, EDTA, *o*-phenanthroline and dipicolinic acid (DPA) due to the arrangement of loosely bound Zn^2+^ ions present in its active site [27]. Then, in 2014, the discovery of a fungal-derived natural product, Aspergillomarasmine A (AMA), was reported as a metal chelator that effectively inhibited NDM-1 and VIM-2 MBLs and thus restored the activity of meropenem [28]. Although promising, recent advances in synthesizing derivatives of AMA could not surpass the biological potency of AMA [29]. Several other reports indicate metal chelators could be rapid and potent MBLIs of the NDM, VIM, and IMP variants. These include the nitrogen donor chelators, ZN148 [30], H_2_dpa [31] and 2-quinazolinone derivatives [32], the sulphonamide-oxygen chelators ANT2681 [33] and *N*-sulfamoylpyrrole-2-carboxylates [34] as well as the 1,2,4-triazole-3-thione compounds bearing 4-ethyl alkyl/aryl sulfide substituents [35].

**Figure 1:**
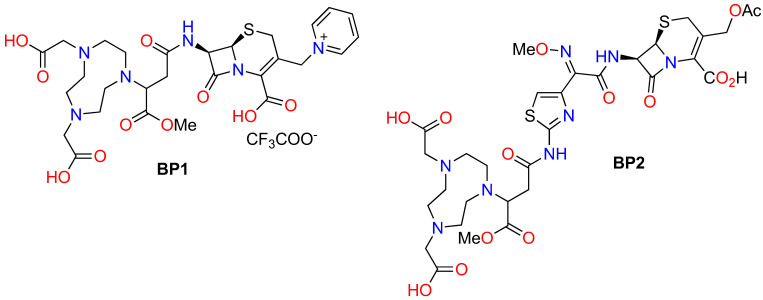
Molecular structure of cyclic amino acidic zinc chelators attached to antibiotic cores.

In some cases, the chelators have been tacked with dipeptide vectors that mimic the bacteria cell wall sequence. However, these have also been shown to possess eukaryotic cell toxicity [36]. Potent chelators would be expected to show off-target effects within the biological environment, thus limiting the therapeutic use of simple zinc binders [1]. Our previous studies indicated that cyclic amino acidic zinc chelators based on contrast agent NOTA exhibit very good β-lactamase inhibition and surprisingly little toxicity; however, the polar nature of NOTA came with pharmacokinetic challenges [37,38,39]. Hence, we envisaged that conjugation of the chelator to a known drug would enhance its properties. This was achieved by attaching a cyclic zinc chelator (NOTA-derived) to known β-lactam antibiotic cores to produce the BP-series of compounds [40] (Figure 1). Based on the excellent results for BP1, herein we expand the scope of the most potent derivative, to BP2, which can also be used as a potential MBLI to restore the efficacy of carbapenems against MBLs.

## 2. Results

The drug susceptibility profile of 21 MBL-expressing bacterial strains were studied against meropenem alone, cyclic amino acidic zinc chelator (BP2) alone, or both compounds in combination. The efficacies of meropenem to 21 MBL expressing bacteria were restored by BP2 (Table 1). The level of synergy exhibited by meropenem and the BP2 compound was also assessed according to the FICI criteria (Table 1). All MBL harboring variants obtained a FICI below 0.5. Serum had no considerable effect on the MIC of the BP2 + meropenem. Furthermore, BP2 displayed specific activity towards MBLs, as there was no activity exhibited against SBL-expressing variants. The overall finding from Table 1 is that BP2 is an effective MBLI against Enterobacterales expressing bla_NDM-1_, bla_NDM-4_, bla_VIM-1_, bla_VIM-2_, bla_VIM-19_, bla_IMP-1_, bla_IMP-8_ and bla_IMP-11_ genes. BP2 successfully restored the meropenem MIC to therapeutically acceptable levels, defined herein as <2 mg/L coupled with a BP2 MIC of <64 mg/L, therefore conforming to the standards outlined by CLSI [41] and ISO 20776-2 [42] regulatory bodies.

**Figure 2:**
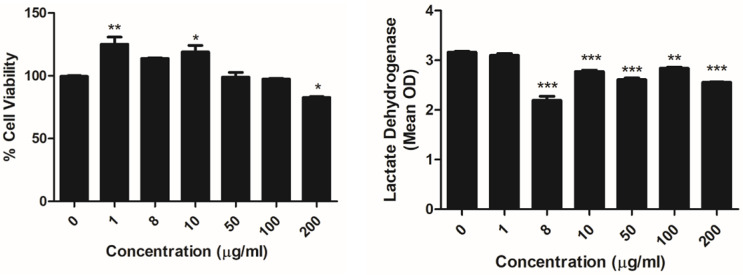
Cell viability studies conducted on HepG2 cells using varying concentrations of BP2. Cell viability was not significantly altered at 50–100 µg/mL; however, it significantly reduced at 200 µg/mL. * *p* < 0.5 and ** *p* < 0.01 relative to control. LDH levels remained unaffected at 1 µg/mL and were significantly reduced at 8–200 µg/mL, indicating BP2 does not induce necrosis in HepG2 cells after exposure. ** *p* < 0.01 and *** *p* < 0.001 relative to control.

BP2 was then investigated for cytotoxicity using a human liver (HepG2) cell line. From the cytotoxicity data, an IC_50_ of 61.98 mg/L was obtained (Figure 2). In general, BP2 (1 µg/mL and 10 µg/mL) significantly increased cell viability compared to the control (Figure 2), whilst at the highest concentration (200 µg/mL), there was a significant decrease in cell viability compared to the control (Figure 2). The Methyl Thiazol Tetrazolium (MTT) assay is a measure cellular metabolic output, and BP2 at low concentrations increases the metabolic output. BP2 is non-toxic to the HepG2 cells at all concentrations tested except 200 µg/mL. HepG2 cell membrane integrity, as measured by Lactate Dehydrogenase (LDH) leakage, was not disrupted at all concentrations tested. In fact, BP2 (8 µg/mL–200 µg/mL) significantly decreased LDH membrane leakage (Figure 2). Except for *S. marcescens* IMP-11 in the checkerboard assay (Table 1), all BP2 concentrations used were 30 mg/L lower than those of the IC_50_. 

**Figure 3:**
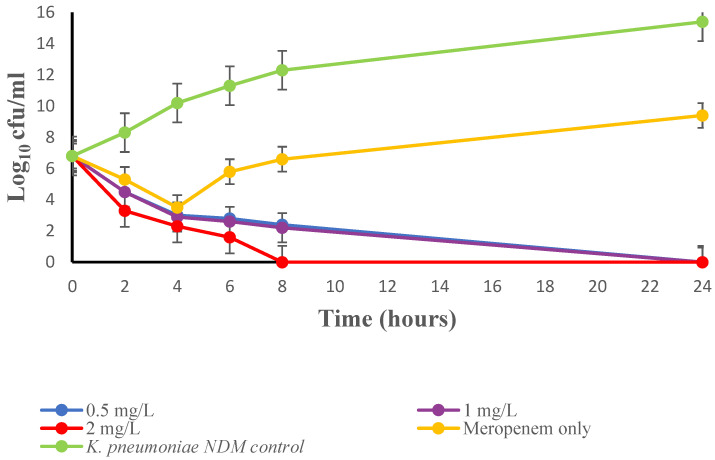
Time-kill kinetic study of *K. pneumoniae* NDM using BP2 MBLI + meropenem. *K. pneumoniae* NDM was challenged with either 2 mg/L (red circles), 1 mg/L (purple circles) or 0.5 mg/L (blue circles) of meropenem + a fixed BP2 concentration of 32 mg/L to ascertain the level of efficacy achieved by combination therapy utilizing differing meropenem concentrations. Combination therapy was compared against monotherapy (meropenem only, yellow circles) and no therapy (green circles, bacterial control).

Figure 3 represents the effect exhibited by BP2 + meropenem over 24 h against the virulent *K. pneumoniae* NDM-expressing bacteria. Excellent bactericidal activity against *K. pneumoniae* NDM with meropenem concentrations of 0.5, 1, and 2 mg/L as well as 32 mg/L BP2 were observed. An appreciable decrease in the bacterial load was observed (>3 log_10_) for each time point relative to the bacterial control. Meropenem monotherapy produced a >2 log_10_ increase in bacteria compared to the BP2 + meropenem-treated group. However, a sharp decrease was observed at 4 h post inoculation for the meropenem control group. This effect was short-lived as the bacteria continued to grow exponentially for the subsequent time points. This was expected as bacterial resistance to meropenem was observed and noted in Table 1.

BP2 was then subjected to enzyme analyses to determine essential enzyme parameters for assessing the level of potency exhibited. The IC_50_ was calculated from the generated sigmoidal curves (Figure S1) using GraphPad Prism software version 8.0.2 (GraphPad Inc., San Diego, CA, USA), and the K*_i_* _app_ was calculated using the Cheng–Prusoff equation [43]. Notably, we report K*_i_* _app_ to compare inhibitors. However, we expect that inhibitors that remove one Zn atom from the enzyme active site will be slow-acting irreversible inhibitors that would require a more detailed kinetic analysis than used here. Table 2 indicates that BP2 is fairly potent against VIM-2, with inhibition very similar to that of BP1 (K*_i_*_app_ = 24.8 µM, previously evaluated by our group); however, BP2 is almost three times more potent with inhibiting NDM-1 than BP1 (K*_i_*_app_ = 97.4 µM). Structurally, BP2 is a thiazole-containing compound, while BP1 lacks this moiety. It is unclear whether the increased inhibition activity is due to the longer distance between the chelator moiety and the lactam moiety, which is less likely to sterically hinder the chelator, or if it is the thiazole itself that boosts the interaction efficacy of the compound.

**Figure 4:**
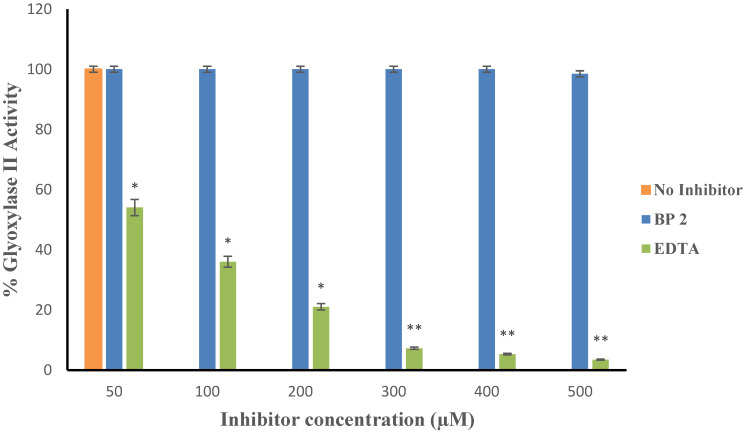
Glyoxylase II activity in the presence of BP2 and EDTA. Normal glyoxylase II activity is denoted by the legend representing no inhibitor added. BP2 did not decrease the activity of glyoxylase II compared to metal chelating agent EDTA. Statistical significance is denoted by *p* < 0.005 (*) and *p* < 0.001 (**) Experiment conducted in triplicate.

Metal chelators are known to suffer from off-target specificity and as a result have been criticized as potential MBLIs in vivo [44]. We designed the following experiment to investigate the level of specificity exhibited by BP2: Recombinant human glyoxylase II (Glo2), an essential zinc-containing enzyme structurally similar to MBLs [45], was monitored for interaction in the presence and absence of BP2. Based on the results from Figure 4, concentrations up to 500 µM of BP2 did not reduce the activity of Glo2, indicating that it does not bind to or remove the zinc ions within the active site of Glo2, whilst metal chelating agent EDTA significantly reduced the activity of Glo2 from 50–500 µM.

**Figure 5:**
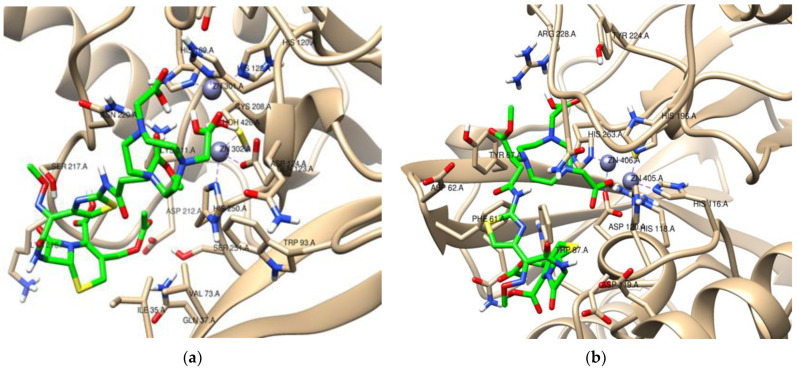
Docked 3D structures of the BP2_SR with NDM-1 and VIM-2 enzyme (shown in green). (**a**) NDM-1-BP2 complex shows the coordination between the chelator carboxylic moieties with Zn^2+^ (301). Zn^2+^ (301) is coordinated to three histidines, and a water molecule (HOH426) that coordinates the two Zn^2+^ ions. Zn^2+^ (302) coordinate histidine, aspartate, and cysteine. (**b**) VIM-2–BP2 complex shows the coordination between the chelator carboxylic oxygen with Zn^2+^ (406). In VIM-2, the Zn^2+^ (405) coordinates three histidines, while Zn^2+^ (406) coordinates histidine, aspartate, and cysteine residues. The chelator carboxylic moieties coordinate Zn^2+^ (405). The diagrams rendered show the BP2_SS configuration. The 3D structures of all the complexes are available with the supplementary material.

Next, we investigated the molecular docking of BP2, followed by a molecular dynamic simulation. This more accurately elucidated the interactions formed and the stability of the conformations were measured over 100 ns in calculations. The ligand interaction diagram (Figure 5) shows that the carboxylic arms of the chelator (which are apparently crucial in the molecular design) remained coordinated to the zinc atom(s) throughout the simulation. This observation implies that the investigated systems can potentially disrupt the binding of these ions in the enzyme active site, as we hypothesized.

The binding energies of the different BP2 configurations and their complexes were determined using MMGBSA post-MD simulations. For both β-lactamases, the model predicts that the BP2_SS configuration has a greater binding free energy than BP2_SR. This is the opposite result to that which we observed in our previous work with BP1 [40]. It should be noted that BP2_SS configuration binds exclusively to a single zinc atom in VIM-2 yielding the largest binding value (Figures S2 and S5). The model implies that enzymatic inhibition may be achieved by affecting the coordination arrangement of only one of the zinc ions in these systems.

**Figure 6:**
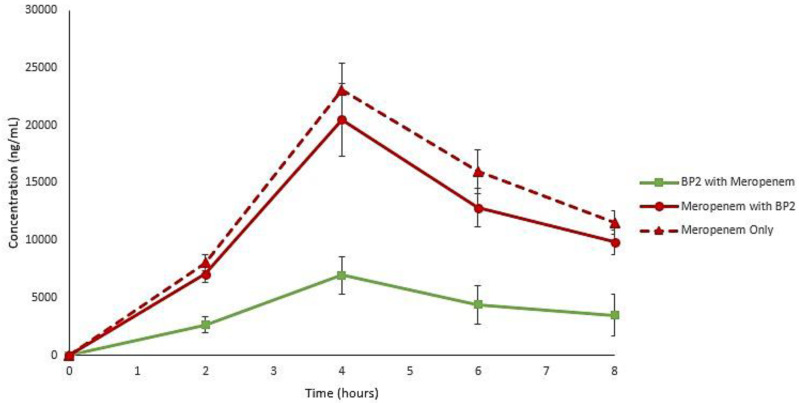
Plasma BP2 and meropenem concentrations over the eight-hour treatment period. BALB/c mice were infected intramuscularly (IM) in the right thigh with 10^6^–10^8^ *K. pneumoniae* NDM cfu/mL. Treatment was initiated two hours post infection with either S, M, or BP2. Four treatment doses were administered via intraperitoneal (IP) injection every two hours. Data are presented as a mean ± SD (*n* = 6). Meropenem-only (red triangle)—animals treated with meropenem only, Meropenem with BP2 (red circles)—meropenem concentration when animals were treated with the BP2 combination. BP2 with meropenem (green square)—concentration of BP2 when animals were treated with the meropenem combination.

In order to evaluate BP2’s potential to act synergetically with antibacterial agents such as meropenem in vivo, we next performed a single-dose pharmacokinetic experiment to ensure an appropriate dosing schedule for achieving a safe and therapeutic concentration of BP2 in plasma. The PK profile between BP2 and meropenem was similar as they obtained peak concentrations (C_max_) 15 min after drug administration. Constant concentrations were then reached after two hours. A plasma concentration of 1.93 µg/L was obtained with a dose of 10 mg/kg.b.w. This was far below the cytotoxicity concentrations seen in the cell viability assays, advocating for the safe increase in the treatment dose to 100 mg/kg.b.w, which allowed us to reach therapeutic concentrations (Figure 6) in plasma without the possibility of toxicity [40]. In vivo efficacy studies were undertaken to assess the potency of BP2 in a murine infection model. This five-day animal trial was initiated by immune-suppressing the mice with cyclophosphamide to allow the progression of infection and allow BP2 + meropenem treatment to be studied over eight hours on day five of the trial. In addition, it excludes the effects of the innate immune response as a potential variable [46]. This is important since many carbapenem-resistant infections are acquired as secondary infections in immune-suppressed hosts [47]. The mice were successfully infected with *Klebsiella pneumoniae* NDM, evidenced by visible inflammation of the localized area; this correlated with the cfu/thigh data expressed (Tables S1–S3). Three treatment regimens of S (saline only), M (meropenem only), and BP2 combination therapy (BP2 + meropenem) were used to randomly categorize mice.

**Figure 7:**
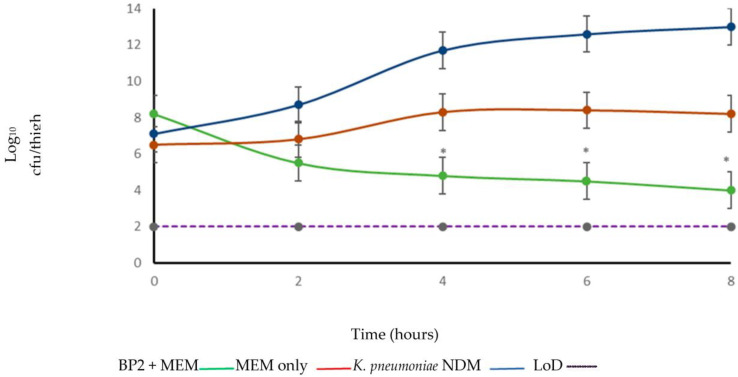
The efficacy of BP2 combination therapy over monotherapy in a murine thigh infection model. Neutropenic BALB/c mice were infected with 0.1 mL of 10^6^–10^8^ cfu of *K. pneumoniae* NDM-1 IM (MEM MIC 128 mg/L). Mice were treated via IP with either S, M (100 mg/kg) or BP2 (100 mg/kg) and meropenem (100 mg/kg) two hours post inoculation. Colony counts in PBS were performed from thigh homogenates every two hours. Treatment groups were analyzed using the ANOVA method of comparison, with a *p* < 0.005 considered statistically significant. The co-administration of BP2 and meropenem resulted in a significant decrease in *K. pneumoniae* NDM cfu/mL in comparison to S and M treatment. This indicates that BP2 + meropenem is a favourable treatment strategy. MEM = Meropenem, LoD = Limit of Detection. *n* = 6 mice per time point per group, cfu/mL counts were done in triplicate. * denotes *p* < 0.005.

Based on previous studies conducted by our research group, we know that BP2 should be administered every two hours with meropenem to account for the short half-life of BP2 and meropenem as well as the rapid renal DHP-1 hydrolysis rate of meropenem [48]. Therefore, a total of four doses (100 mg/kg of each drug) over eight hours were administered with no visible observations of toxicity (total drug dose 800 mg/kg). Treatment was restricted to eight hours to ensure the trial was logistically viable. Moreover, injecting an animal of that size repeatedly is unethical and does not concur with the principles of ARRIVE used to guide the trial’s design. The results from Figure 7 indicate that although BP2 is more potent in vitro, it shares a similar in vivo efficacy to our previously reported cyclic zinc chelator covalently attached to a cephalosporin.

Figure 7 depicts the outcome of a successful murine infection model. *K. pneumoniae* NDM colonies were reduced by >3 log_10_ units, and the deviation between the doses of BP2 + meropenem administered was <8% (Tables S1—S3, indicating statistical significance (*p* < 0.005).

## 3. Discussion

The utilization of metal chelators in combination therapy to target Carbapenem-Resistant Enterobacterales proves to be a promising treatment strategy as observed in reports of di- and tris-picolylamine zinc chelators [49,50,51]. Our previous results also indicated this, that is, with BP1 [40]. This led us to investigate other derivatives of BP1 (cyclic amino acidic zinc chelators attached to a β-lactam moiety) to study other aspects for optimization of the derivatives/hits with regards to synthetic route viability, solubility, affinity, selectivity, efficacy/potency, metabolic stability, and oral bioavailability. The results from the drug susceptibility assay (Table 1) indicate that virulent strains of NDM-producing bacteria were observed to be highly resistant to meropenem monotherapy but susceptible to combination therapy (Table 1). In combination therapy, the BP2 compound was highly potent in restoring the efficacy of meropenem to concentrations ≤ 1 mg/L for 21 MBL variants. When comparing the biological activity of the BP2 inhibitor reported herein to other metallo-β-lactamase inhibitors, the MICs generated by BP2 are highly efficacious. These observations further indicate that BP2 is superior to the concentrations reported by Ishii et al. for the malic acid derivative ME1071 [52] and Everett et al. for ANT431 [48]. When comparing our results to the nitrogen donor chelators that we believe are mechanistically similar MBLIs [30,31], the reported activities concur. However, BP2 exhibited better efficacies with lower MBLI concentrations. Of note is the high level of synergism exhibited, with a FICI of ≤0.14 for meropenem and BP2 during co-administration against the 21 bacterial isolates studied. When evaluating the safety profile of BP2, cytotoxicity investigations proved the chelator to be non-toxic at the utilized concentrations and aids in increasing HepG2 metabolic output (Figure 2). The time-kill study (Figure 3) indicated that BP2 co-administered with meropenem produced bactericidal activity of >3 log_10_ units over 24 h against *K. pneumoniae* NDM. Similar bactericidal trends of a 3 log_10_ bacterial reduction were found with BP1 and the h_2_dpa derivatives [31]; however, h_2_dpa derivatives utilized a concentration two-fold lower than that of BP2 and achieved a bacterial reduction to 10 cfu/mL, whilst BP2 achieved complete killing. When comparing our time-kill results to ZN148, BP2 and meropenem were both utilized at half the required quantity of ZN148 and meropenem, and were, therefore, superior in the drug susceptibility assessment [30]. Reports from pyridyl type chelators co-administered with meropenem are concordant with this study, since similar bactericidal trends of ≥3 log_10_ reduction in the growth of MBL-producing bacteria were observed experimentally [53]. These findings are characteristic of β-lactams and indicate that meropenem’s efficacy has been restored due to the co-administration of BP2. No observations of bacterial re-growth were observed as complete killing was achieved, as illustrated by Figure 3. The results from our study indicate that combination therapy between BP2 and meropenem produces a bactericidal effect by a measure of ≥4 log_10_ cfu/mL over a 24 h period and at meropenem concentrations as low as 0.5 mg/L for the BP2 chelator investigated (Figure 3). Further investigation of BP2 indicated inhibitor-specific activity towards the NDM-1 and VIM-2 MBLs, as there was no reduction in glyoxylase activity. These findings correspond to MBLI chelators’ reports, BP1 [40] and ZN148 [30]. It is possible that BP2 ultimately removes the zinc ions from the active site, immobilizing the enzyme completely (Figure 5). This is supported by the absence of a MIC value when NOTA pre-complexed to zinc was evaluated as a potential MBL inhibitor (Table 1), confirming that BP2′s zinc chelation is required for inhibition. Subsequent studies will use PACs-MD [54] to determine whether NOTA chelation of the zinc ion is energetically feasible. Many MBLI candidates produce good in vitro efficacies but fail to reach efficacy in vivo. These include NOTA [37] with poor bioavailability and TPEN [38] with cytotoxicity [55], both previously researched by our group. The data generated in Figure 7 clearly indicates the success of combination therapy in a murine infection model. Based on the extrapolation of the treatment curve of Figure 7, a further decline in the cfu/thigh count would have been observed with continued treatment. However, considering the animal’s welfare, we could not risk the fate of severe inflammation/animal demise. Based on the results (Figure 7), the in vivo activity of BP2 is concordant with the in vivo efficacy of BP1 and ZN148, where a decrease in the bacterial load is observed to a count of approximately 3 log_10_ units [30], thus implying that BP2 is efficacious in restoring the potency of meropenem.

## 4. Materials and Methods

### 4.1. Synthesis of MBLIs

The Catalysis and Peptide Research Unit of the University of Kwa-Zulu Natal, Westville, Durban, South Africa synthesized and characterized the BP2 chelator.

#### Synthesis of BP2

**Scheme 1:**
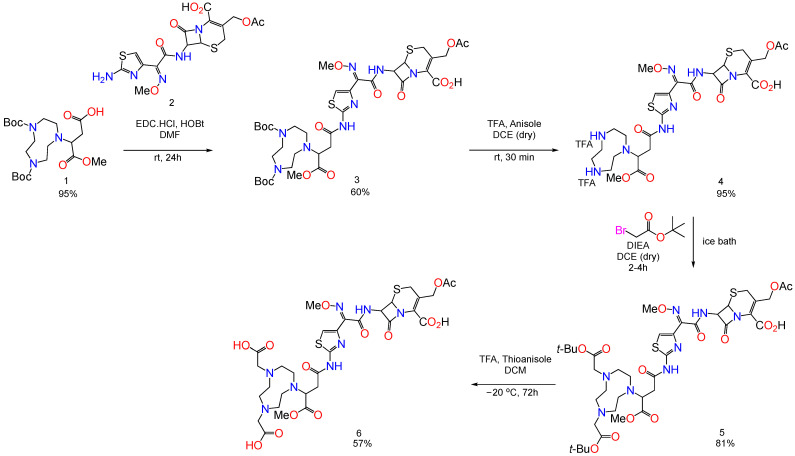
Synthetic route to BP2.

As per Scheme 1, 4-Methoxy-1,4,7-triazacyclononane-butanoic acid analogue (1) was coupled with a commercially available cephalosporin (2) using the peptide coupling agent EDC to furnish (3) in 60% yield in its racemic form. The Boc protecting groups were then removed with TFA to yield 95% of compound (4). Thereafter, the amines on the cyclononane were alkylated (5), and the t-butyl protecting groups were subsequently removed using trifluoroacetic acid (TFA) to produce the de-protected final product (6 aka BP2) in 57% yield as an off-white solid. All reactions were monitored and optimized using liquid chromatography-mass spectrometry (LC-MS), and products were fully characterized using standard methods.

### 4.2. Bacterial Source

CRE strains producing MBLs were acquired from Patrice Nordmann at the Institut National de la Santé et de la Recherche Médicale (U914), Paris, France [56]. The bacterial strains used included *E. coli*, *E. cloacae, S. marcescens*, *P. rettgeri*, *K. pneumoniae*, and *P. stuartii* variants harbouring MBLs. *K. pneumoniae* NDM was acquired from David P. Nicolau at the Center for Anti-Infective Research and Development, Hartford Hospital, USA [57]. An *E. coli* ATCC 25922 was employed as a carbapenem-susceptible control. All bacterial stock solutions were preserved in Trypticase soy broth and 10% glycerol containing 4 mm glass beads at −80 °C. Meropenem was obtained from Sigma-Aldrich (Schnelldorf, Germany), and BP2 compounds were synthesized and characterized by the Catalysis and Peptide Research Unit, University of KwaZulu Natal, South Africa. Meropenem was prepared in distilled water (*m*/*v*), and the BP2 compound was prepared in 50% (*m*/*v*) DMSO. The final DMSO concentration was <1.0%. Antimicrobial stock solutions were stored at −80 °C.

### 4.3. Drug Susceptibility Testing

#### 4.3.1. Broth Microdilution Assay

The Minimum Inhibitory Concentration (MIC) of meropenem BP2 was determined utilizing the broth microdilution assay, as described by the Clinical and Laboratory Standards Institute (CLSI) antimicrobial susceptibility guidelines [58]. The mono-therapeutic effect of each antibiotic and MBLI was evaluated across a panel of MBL-producing Enterobacterales.

#### 4.3.2. Checkerboard Assay

The drug susceptibility profile of meropenem in combination with BP2 was studied using the checkerboard assay. This assay was used to ascertain the effect of two drugs on antimicrobial resistance targeting CRE strains. The assay was performed according to the protocol described previously [59] and was under CLSI antimicrobial susceptibility guidelines [58]. Briefly, twofold dilutions of meropenem with each chelator were made in Cation-adjusted Mueller Hinton Broth (CAMHB) in a 96-well microtiter plate. A 0.5 McFarland-standardized bacterial inoculum was added to each well to obtain a final volume of 100 µL per well. Thereafter, plates were incubated at 37 °C for 18 h under aerobic conditions. The checkerboard assays were performed in triplicate. The MIC was determined as the lowest concentration at which no visible growth was present. The fractional inhibitory concentration index (FICI) was calculated for each combination according to the equation FICI = FIC_a_ (MIC of drug A in combination/MIC of drug A alone) + FIC_b_ (MIC of drug B in combination/MIC of drug B alone) [60]. The FICI was interpreted as follows: synergy, FICI ≤ 0.5; additive 0.5–1; indifference, >0.5 FICI < 4; and antagonism, FICI ≥ 4 [61].

#### 4.3.3. Effects of Human Serum

To study the effects of human serum on the MIC values, the above antimicrobial susceptibility testing protocol was adopted. However, the broth was prepared differently. MHB was prepared according to the manufacturer’s instructions (Oxoid Ltd., ThermoFisher Scientific, Hampshire, United Kingdom). Thereafter, equal volumes of broth and 100% human serum were utilized to generate a medium that contained 50% human serum.

#### 4.3.4. Time-Kill Study

Time-kill studies were performed according to previously published methods [53], including those described by CLSI document M26-A [62]. Briefly, an overnight culture of *K. pneumoniae* NDM was diluted to a 0.5 McFarland standard that correlated to approximately 10^6^ cfu/mL. The prepared bacterial suspensions were added to 96-well plates containing a fixed dose of 32 mg/L of BP2 and meropenem in concentrations of 0.5, 1 or 2 mg/L. Plates were incubated at 35 °C and 100 rpm shaking. A bacterial control without adding any drugs was included, and a meropenem-only control employing similar conditions. Viability counts were performed at 0, 2, 4, 6, 8, and 24 h by sampling 0.1 mL, diluting as appropriate, and spreading onto Mueller Hinton agar (MHA). These plates were incubated at 35 °C for at least 18 h. Colonies were enumerated as cfu/mL.

### 4.4. Cytotoxicity Assay

#### 4.4.1. Cell Culture

HepG2 cells were cultured in 25 mL cell culture flasks using Eagle’s minimum essentials medium (EMEM) supplemented with 10% fetal bovine serum, 1% pen-strep-fungizone and 1% L-glutamine, maintained in a humidified incubator (37 °C, 5% CO_2_) until approximately 80% confluence.

#### 4.4.2. MTT Assay

The MTT assay was one of the methods used to determine in vitro cell viability of BP on HepG2 cells. HepG2 cells (15,000 cells/well) were seeded into a 96-well microtiter plate and allowed to adhere overnight (37 °C, 5% CO_2_). Thereafter, the cells were incubated (37 °C, 5% CO_2_) with a range of BP concentrations (0, 1, 8, 10, 50, 100 and 200 μg/mL) in triplicate for 6 h. After the 6 h incubation, the cells were washed with 0.1 M phosphate-buffered saline (PBS) and incubated with MTT salt solution (5 mg/mL in 0.1 M PBS) and 100 μL CCM for 4 h (37 °C, 5% CO_2_). The MTT salt solution was removed, and DMSO (100 μL/well) was added and incubated for 1 h. The optical density was measured using a spectrophotometer (Bio-Tek μQuant) at 570/690 nm. Results are expressed as % cell viability versus BP2 concentration (μg/mL).

#### 4.4.3. LDH Assay

The LDH assay was used to assess membrane damage of HepG2 cells. Supernatant collected from control and BP2 treated cells were centrifuged (400× *g*, 24 °C, 10 min) and dispensed (100 μL/well) in triplicate into a 96-well microtiter plate. LDH reagent (100 μL, 11644793001, Sigma Aldrich, Schnelldorf, Germany) was added to each well. The plate was incubated for 30 min at room temperature in the dark. Absorbance was read using a spectrophotometer (Bio-Tek μQuant,) at 500 nM. Results are represented as relative fold change compared to untreated control.

### 4.5. Enzyme Assays

#### 4.5.1. Inhibition of Kinetics

A dose-dependent enzyme inhibition assay was performed using a Biotek PowerWave XS2 (Biotek Instruments Inc., Winooski, VT, USA) plate reader. NDM-1 and VIM-2 enzymes were purchased from RayBiotech (RayBiotech Life Inc., Peachtree Corners, GA, USA). Enzymes in the quantity of 1 nM (NDM-1) or 0.5 nM (VIM-2) were used in this study with a fixed nitrocefin concentration of either 120 µM (NDM-1) or 50 µM (VIM-2) and varying BP2 concentrations ranging from 5 to 200 µM (NDM-1) and 2.5 to 300 µM (VIM-2) in 50 mM HEPES buffer supplemented with 100 µg/mL BSA and 10 µM ZnCl_2_. Inhibition was measured at 482 nm at 25 °C.

#### 4.5.2. Non-Specific Binding of the Inhibitor to Zinc in Non-MBLs

To determine the binding specificity of the inhibitors to other zinc-containing enzymes, glyoxylase (BioVision Inc., Waltham, MA, USA) was utilized to measure the level of specificity exhibited by BP2. The methodology has been previously described [30]. EDTA (purchased from Merck KGaA, Darmstadt, Germany) was included as a positive control.

### 4.6. Ethical Statement

All animal experiments carried out in this study were approved by the Institutional Animal Research Ethics Committee at the University of KwaZulu-Natal, with approval reference AREC/00002618/2021 (for the in vivo efficacy study). All sample sizes used in this study were estimated using G*Power Version 3.1.9.4.

### 4.7. In Vivo Efficacy Study

A murine thigh infection model described by Michail et al. [63] was performed with minor modifications. Male Bragg inbred albino c-strain (BALB/c) mice weighing 20–25 g (*n* = 90) were used in this study. Each of the three groups constituted *n* = 30 mice, with *n* = 6 mice receiving treatment every two hours. Prior to infection, mice were IP treated with 150 mg/kg cyclophosphamide on day one and 100 mg/kg on day four of the trial. This was performed to induce neutropenia in the mice. Neutropenia was confirmed by a neutrophil count of <100/mm^3^. On day five of the trial, 0.1 mL inoculum containing 10^6^–10^8^ cfu/mL of *K. pneumoniae* NDM were IM injected into the right thigh of the mice to initiate infection. Meropenem monotherapy, BP2 + meropenem combination therapy, or normal saline was administered every two hours in an eight-hour treatment period. Mice were euthanized by isoflurane overdose at 2 h, 4 h, 6 h and 8 h post dosing. The right thigh muscle was then aseptically removed and homogenized in 5 mL PBS. Homogenates were spread onto Mueller–Hinton agar and MacConkey agar plates, followed by incubation at 35 °C for 24 h and enumeration of the cfu/mL.

### 4.8. LC-MS Quantification

A Shimadzu Nexera Series (Shimadzu Corporation, Kyoto, Japan) liquid chromatography system was coupled with Shimadzu LCMS-8050 tandem mass spectrometer (Shimadzu, Kyoto, Japan). The chromatographic separation was achieved using a Shim-Pack Velox SP-C18 column (100 mm × 2.1 mm, 2.7 µm particle size) with a gradient mobile phase comprised of Millipore water (0.1% *v*/*v* trifluoroacetic acid) (A) and Acetonitrile (0.1% *v*/*v* trifluoroacetic acid) (B). The gradient method started from 5 to 95% B in 8 min, then held at 95% B for up to 12 min; thereafter, it was brought back to 5% B at 12.1 min. The column was equilibration time was 2.9 min with a flow rate of 0.4 mL min^−1^ and the column oven temperature at 40 °C. The injection volume was 25 µL, and the total run time of the method was 15 min. Quantitative and qualitative studies were conducted using MRM mode via an ESI interface, with the following source parameters: nitrogen nebulizer gas flow 3 L/min; heat gas 10 L/min and interface temperature of 300 °C. The precursor and product ions optimized were *m*/*z* 813.2→330.0 for BP2, *m*/*z* 384.50→68.25 for meropenem and *m*/*z* 350.50→304.40 for Ampicillin (IS). Results were analyzed using LabSolutions Insight LCMS. All data are expressed as a mean ± SD.

### 4.9. Computational Studies

These methods have been detailed in the supplementary information (pages S6–S8).

### 4.10. Statistical Analyses

GraphPad Prism version 8.0.2 (GraphPad Inc., San Diego, CA, USA) was utilized to analyze the data generated from the time-kill assay, as well as the enzyme inhibition assays. The rate at which combination therapy resulted in bactericidal activity was determined per time point using a linear regression model. The two-way analysis of variance (ANOVA) was used in the in vivo efficacy study, the three treatment groups, S (saline only), M (meropenem monotherapy) and BP2 and meropenem combination therapy (BP2 and meropenem) were compared. Statistical significance was represented by a decrease in the *p*-value (*p* < 0.05) and an increase in the F ratio.

## 5. Conclusions

The cyclic amino acidic zinc chelator, BP2, described in this study functions as a promising MBLI by restoring the efficacy of meropenem to susceptible antimicrobial concentrations against various MBL-expressing bacteria. Very few MBLIs have the potential to restore meropenem to such low concentrations without causing an adverse effect to the eukaryotic cells. Furthermore, the co-administration of meropenem with BP2 has proven to have a synergistic effect across a panel of MBL-harboring bacteria. Analyses from the time-kill experiments also indicated that the metal chelator displayed a bactericidal effect on *K. pneumoniae* NDM. BP2 restored meropenem’s potency, facilitating the 24 h killing of *K. pneumoniae* NDM at all concentrations tested. In addition, bacterial re-growth was absent as complete killing was achieved. Given the successful results obtained from the in vitro tests, BP2 was pursued further with in vivo experiments. BP2 showed activity against *K. pneumoniae* NDM-1 infection in a murine study when combined with meropenem. The overall findings of this study indicate that BP2 is a promising therapeutic lead for targeting MBL-mediated carbapenem resistance, making this class of compounds worthy for further pre-clinical assessment.

## 6. Patents

BKP, HGK, PIA, TN, and TG have a patent on the technology [64].

## Figures and Tables

**Table 1:** The MICs of BP2 and meropenem alone or in combination, from a panel of 21 carbapenem-resistant bacteria.

Bacterial Reference	Bacterial Strain	MBL Produced	MIC (mg/L)			FICI
			MEM Alone	BP2 Alone	BP2 + MEM	
ATCC 25922	*Escherichia coli*	N/A	0.06	N/A	0 + 0.06	N/A
AUS 271	*Escherichia coli*	NDM-1	>128	>256	16 + 0.25	0.13
FEK	*Escherichia coli*	NDM-4	>32	>256	16 + 0.5	0.07
JAP	*Escherichia coli*	IMP-1	8	>256	16 + 0.03	0.04
TWA	*Escherichia coli*	IMP-8	8	>256	32 + 0.06	0.09
BM 14	*Escherichia coli*	VIM-1	32	>256	8 + 0.25	0.04
TC CARF	*Escherichia coli*	VIM-2	8	>256	4 + 0.5	0.08
DIH	*Escherichia coli*	VIM-19	>32	>256	16 + 0.125	0.07
IR 386	*Enterobacter cloacae*	NDM-1	32	>256	16 + 0.125	0.07
BM 5	*Enterobacter cloacae*	IMP-1	>32	>256	32 + 0.5	0.14
TWA	*Enterobacter cloacae*	IMP-8	0.125	>256	16 + 0.06	0.5
KAR	*Enterobacter cloacae*	VIM-1	4	>256	16 + 0.25	0.13
USA 449	*Klebsiella pneumoniae*	NDM	>32	>256	16 + 0.25	0.07
6852	*Klebsiella pneumoniae*	IMP-1	>32	>256	64 + 1	0.3
TWA	*Klebsiella pneumoniae*	IMP-8	8	>256	16 + 0.25	0.09
ENNES	*Klebsiella pneumoniae*	VIM-1	>32	>256	8 + 0.5	0.05
DIH	*Klebsiella pneumoniae*	VIM-19	32	>256	16 + 0.125	0.07
	*Serratia marcescens*	IMP-11	>32	>256	64 + 1	0.3
BM 20	*Serratia marcescens*	VIM-2	>32	>256	8 + 0.5	0.05
PSTU	*Providencia stuartii*	NDM-1	32	>256	8 + 0.25	0.04
IR38	*Providencia Rettgeri*	NDM-1	32	>256	32 + 0.5	0.14

MEM = Meropenem, N/A = Not applicable. All assays were conducted in triplicate. Synergy, FICI < 0.5; additive 0.5–1; indifference, >0.5 FICI < 4; and antagonism, FICI > 4. Compound (2) i.e., the cephalosporin component of BP2, displayed no activity on its own. NOTA pre-complexed to zinc displayed no activity towards the MBL harboring bacteria. BP2 + meropenem did not show activity towards *S. marcescens* KPC-2 and *E. coli* OXA-28, indicating BP2 has specific activity towards MBLs.

**Table 2:** Enzyme inhibition exhibition by BP2.

MBL	IC_50_ (µM)	K*_i_* _app_ (µM)
NDM-1	70.7 ± 4.5	35.3
VIM-2	57.1 ± 2.9	30.9

All assays were conducted in triplicate. IC_50_ represents the mean ± SD. K*_i_* app was determined using the Cheng–Prusoff equation.

## Data Availability

The data presented in this study are available within the article or supplementary material.

## Supplementary Materials

The following supporting information can be downloaded at: https://www.mdpi.com/article/10.3390/antibiotics12040633/s1, Figure S1: Half the maximal inhibitory concentration of BP2 (A), NOTA (B) and TPEN (C) against NDM-1 and VIM-2; Table S1: *Klebsiella pneumoniae* NDM-infected mice receiving the placebo; Table S2: *Klebsiella pneumoniae* NDM-infected mice receiving meropenem only treatment; Table S3: *Klebsiella pneumoniae* NDM-infected mice receiving BP2 and meropenem treatment; Table S4: Summary of plasma drug concentrations from the in vivo efficacy study; Figure S2. Interaction diagrams of the investigated systems (a) NDM-1—BP2_SR, (b) NDM-1—BP2_SS, (c) VIM-2—BP2_SR, and (d) VIM-2—BP2_SS; Table S5: Docking scores and the binding free energies for NDM-1—BP2 and VIM-2—BP2 complexes; Figure S3: RMSD plot for NDM-1 Ca-backbone aligned with BP2_SR; Figure S4: RMSD plot for NDM-1—BP2_SS complex; Figure S5: RMSD plot for VIM-2—BP2_SR complex; Figure S6: RMSD plot for VIM-2—BP2_SS complex and spectra. Synthetic and computational methods have also been described. [65,66,67,68,69,70,71,72,73,74,75,76,77]
