# Supplementary material for: Neutralizing Carbapenem Resistance by Co-Administering Meropenem with Novel β-Lactam-Metallo-β-Lactamase Inhibitors
Source: Antibiotics (Basel). 2023 Mar 23;12(4):633. doi: 10.3390/antibiotics12040633 (PMC10135050; doi:10.3390/antibiotics12040633)
Supplement: Supplementary file 1 [file antibiotics-12-00633-s001.zip › antibiotics-2276896-SI.pdf]

Appendix A-Supplementary Information: Methodology and Results

Neutralizing carbapenem resistance by co-administering meropenem with novel β-lactam-metallo-β-lactamase inhibitors

Nakita Reddy[1], Letisha Girdhari[1], Mbongeni Shungube[1], Arnoldus C. Gouws[1], Byron K. Peters[1], Kamal K. Rajbongshi[1], Sooraj Baijnath[1, 2], Sipho Mdanda[1], Thandokuhle Ntombela[1,] Thilona Arumugam[3], Linda A. Bester[4], Sanil D. Singh[5], Anil Chuturgoon[3], Per I. Arvidsson[1,6], Glenn E. M Maguire[1,7], Hendrik G. Kruger[1], Thavendran Govender[8*] and Tricia Naicker[1*]

1. Catalysis and Peptide Research Unit, University of KwaZulu Natal, Durban, 4001, South Africa
2. School of Physiology, Faculty of Health Sciences, University of the Witwatersrand, Johannesburg, Gauteng, 2193, South Africa
3. School of Laboratory Medicine and Medical Sciences, College of Health Sciences, University of KwaZulu-Natal, Durban, South Africa
4. Biomedical Research Unit, School of Laboratory Medicine and Medical Sciences, College of Health Sciences, University of KwaZulu-Natal, Durban 4000, South Africa
5. Department of Pharmaceutical Sciences, University of KwaZulu-Natal, Westville Campus, Durban 3629, South Africa
6. Science for Life Laboratory, Drug Discovery & Development Platform & Division of Translational Medicine and Chemical Biology, Department of Medical Biochemistry and Biophysics, Karolinska Institutet, Stockholm, Sweden
7. School of Chemistry and Physics, University of KwaZulu Natal, Durban, 4001, South Africa
8. Department of Chemistry, University of Zululand, Private Bag X1001, KwaDlangezwa 3886, South Africa

*Corresponding authors': naickert1@ukzn.ac.za or govendert@unizulu.ac.za

Methods..3

Synthesis of BP 2..3

Computational Methods..6

Figures, Tables and Spectra..9

Figure S1: Half the maximal inhibitory concentration of BP 2 (A), TPEN(B) and NOTA (C) against NDM-1 and VIM-2. TPEN and NOTA were used as positive controls in the assay........9

Table S1: *Klebsiella pneumoniae* NDM infected mice receiving the placebo. 9

Table S2: *Klebsiella pneumoniae* NDM infected mice receiving meropenem only treatment....10

Table S3: *Klebsiella pneumoniae* NDM infected mice receiving BP 2 and meropenem treatment..10

Table S4: Summary of plasma drug concentrations from the *in vivo* efficacy study 10

Figure S2. Interaction diagrams of the investigated systems (a) NDM-1—BP 2_SR, (b) NDM-1—BP 2_SS, (c) VIM-2—BP 2_SR, and (d) VIM-2—BP 2_SS 11

Table S5: Docking scores and the binding free energies for NDM-1—BP 2 and VIM-2—BP 2 complexes 12

Figure S3: RMSD plot for NDM-1 Ca-backbone aligned with BP 2_SR 12

Figure S4: RMSD plot for NDM-1—BP 2_SS complex 13

Figure S5: RMSD plot for VIM-2—BP 2_SR complex 14

Figure S6: RMSD plot for VIM-2—BP 2_SS complex 14

Spectra 15

Reference 25

Methods

Synthesis of BP 2

Cefotaxime was purchased from Sigma Aldrich (Germany), DLD Scientific (South Africa) and Hangzhou Dayangchem Co., Ltd (China). Reagents and solvents were purchased from Sigma Aldrich and Merck. All solvents were dried by means of standard procedures. Thin Layer Chromatography (TLC) was performed using Merck Kieselgel 60 F254 plates. The synthetic steps were monitored using LC-MS (Shimadzu 2020 UFLC-MS, Japan). The LC-MS method used a gradient of 5% ACN: H2O (0.1% formic acid) to 95% ACN:H2O (0.1% formic acid) over 9 minutes on an XBridgeTM C18 5um 4.6x150mm column, where the flow rate is 1mL/min. Purification of the intermediates was done by gravity column chromatography (mesh particle size, 40-63 µm). High resolution mass spectrometric data were obtained with a Bruker micrOTOF-Q II instrument that operated at ambient temperatures and at a sample concentration of 2 µg/ml. Infrared spectrometric data were recorded on a Perkin Elmer spectrum 100 instrument with a universal ATR attachment. NMR data were recorded using a Bruker AVANCE III 400 MHz at room temperature. Chemical shifts are expressed in ppm and coupling constants are reported in Hz.

Note for the NMR spectra of the final compounds:

The NMR spectra of the final compounds appeared with many overlapping signals due to conformers. This was in particular, attributed to the presence of rotamers and/or the ability of the chelator moieties to appear bent due to the 3D structure of the lactam (four membered ring adjacent to five/ six membered ring).[1] The flexible nature of the molecule is likely to result in broadening of the signals leading to the poor resolution of the multiplets. This is further complicated by overlapping signals arising from the many protons in similar environments. The signal integration corresponded to the number of protons on the products however the spectra appeared 'messy' due to this overlap. After consulting with our collaborators in Sweden, it was decided to subject the samples to temperature variation experiments as well as complexing the chelators with either Zn or Cu. No significant changes in the spectra however were observed (in their laboratories).[2] Hence the NMR spectra of all starting material was recorded and confirmed. The signals of the NMR spectra of all chelators on their own also displayed a significant degree overlap. For these reasons, the final compounds were further

treated and characterised as per peptides in organic synthesis in which the NMR spectra are not recorded however supported by other means of characterisation.[3]

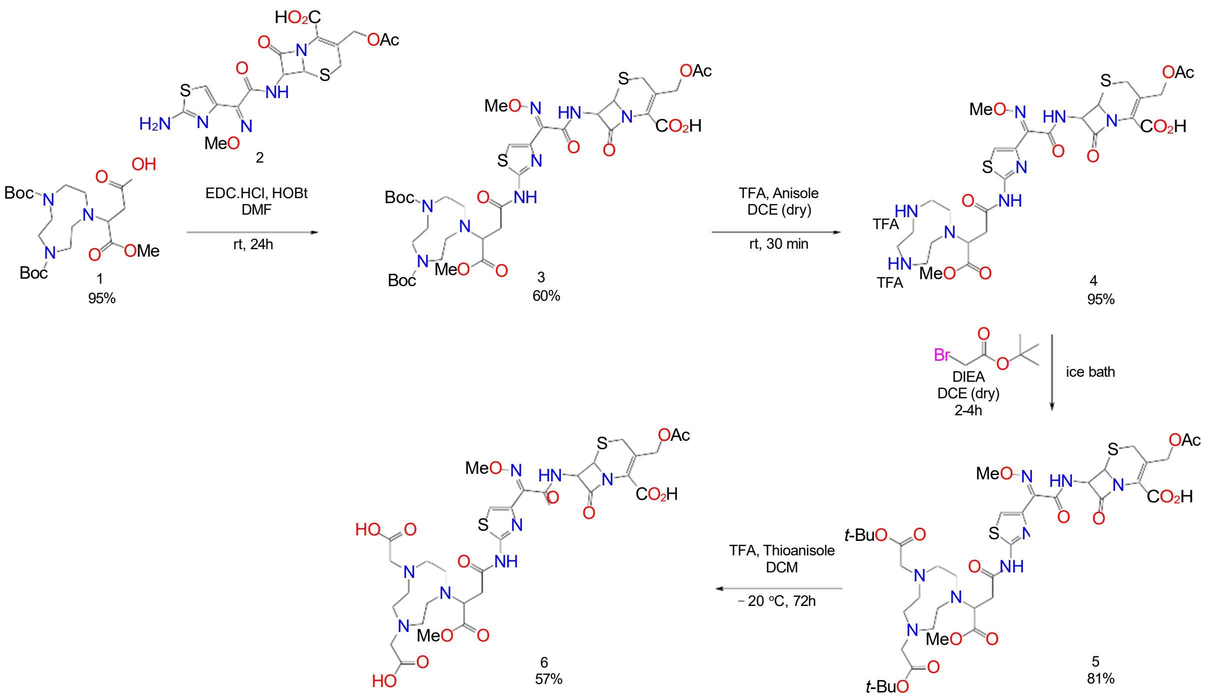

Scheme 1: Synthetic route of BP2

(*E*)-3-(Acetoxymethyl)-7-(2-(2-(3-(4,7-*bis*(*tert*-butoxycarbonyl)-1,4,7-triazonan-1-yl)-4-methoxy-4-oxobutanamido)thiazol-4-yl)-2-(methoxyimino)acetamido)-8-oxo-5-thia-1-azabicyclo[4.2.0]oct-2-ene-2-carboxylic acid (3)

Compound **1** (1.0 g, 2.17 mmol) was dissolved in DMF (2.20 mL, 1.0 mL/mmol) followed by the coupling agent HOBt (352.5 mg, 1.2 equiv.), EDC.HCl (500.0 mg, 1.2 equiv.). The mixture was stirred for 15 minutes (activation period) followed by the addition of cefotaxime (**2**) (990.2 mg, 1.0 equiv.). The reaction was stirred overnight (or longer – depending on conversion - monitored with LC-MS) at room temperature. The product was extracted three times with DCM and water, aided by centrifugation to separate the resulting emulsion, then the DCM layer was extracted with brine. The organic layer was dried over anhydrous magnesium sulphate and concentrated to dryness *in vacuo*. Yields were 40-60 %

A brown, thick oil was obtained with a yield of 60%. Confirmed with LC-MS, m/z = 897 $(m+H)^+$. IR ((υmax/cm^{-1}) . 2974, 2938, 2359, 1754, 1732, 1667, 1539, 1461, 1414, 1367,

1247, 1200, 1142, 1034, 988, 855, 774. HRMS (ESI+) m/z $(m+H)^+$ calculated for $C_{37}H_{52}N_8O_{14}S_2$: 897.3189; found: 897.3245

(*E*)-3-(Acetoxymethyl)-7-(2-(2-(4-methoxy-4-oxo-3-(1,4,7-triazonan-1-yl)butanamido)thiazol-4-yl)-2-(methoxyimino)acetamido)-8-oxo-5-thia-1-azabicyclo[4.2.0]oct-2-ene-2-carboxylic acid (4)

Compound **4** (300 mg, 0.33 mmol) was dissolved in DCE (3.40 mL, 8.0 mL/mmol) followed by the addition of anisole (0.37 mL, 8.0 equiv.) then TFA (2.6 mL, 6.0 mL/mmol). The reaction was stirred at room temperature for 1 hr. Once the reaction was complete the TFA was removed under a stream of nitrogen in the fume hood and washed three times with cold diethyl ether (~ 3.0 mL). The precipitate was isolated using centrifugation and the pellet was dried under a gentle stream of argon gas, until it was a free-flowing powder. A pale-yellow, powder was obtained with a yield of 95% (142 mg).

Confirmed with LC-MS, m/z = 697 $(m+H)^+$. IR (υmax/cm^{-1}) 3712, 3674, 3269, 2981, 2865, 2711, 1726, 1678, 1664, 1637, 1607, 1544, 1491, 1178, 1154, 1129, 1053, 834, 721 HRMS (ESI+) m/z $(m+H)^+$ calculated for $C_{27}H_{36}N_8O_{10}S_2$: 697.2076; found: 697.2154

(*E*)-3-(Acetoxymethyl)-7-(2-(2-(3-(4,7-*bis*(2-(*tert*-butoxy)-2-oxoethyl)-1,4,7-triazonan-1-yl)-4-methoxy-4-oxobutanamido)thiazol-4-yl)-2-(methoxyimino)acetamido)-8-oxo-5-thia-1-azabicyclo[4.2.0]oct-2-ene-2-carboxylic acid (5)

Compound **5** (250 mg, 0.35 mmol) was dissolved in DCM/DCE (2.5mL, 5.0 mL/mmol) and DIEA (0.94mL, 20 equiv.) followed by the addition of tertbutyl bromoacetate (1.5mL, 2.05 equiv.). The reaction was stirred at 0°C to room temperature for 3 hrs while being monitored by LC-MS for completion. The solvent was evaporated and excess DIEA was easily decanted the product was washed with cold diethyl ether (~ 3.0 mL). The precipitate was isolated using centrifugation and the pellet was dried under a gentle stream of argon gas, until it was a free-flowing powder. A pale yellow, thick oil was obtained with a yield of 41% (67 mg).

Confirmed with LC-MS, m/z = 926 $(m+H)^+$. IR (υmax/cm^{-1}), 2936, 2865, 2360, 2343, 1730, 1671, 1544, 1393, 1364, 1057, 983, 833, 798, 746, 681. HRMS (ESI+) m/z $(m+H)^+$ calculated for $C_{39}H_{56}N_8O_{14}S_2$: 926.3436; found: 926.3560

(*E*)-2,2'-(7-(4-((4-(2-((3-(acetoxymethyl)-2-carboxy-8-oxo-5-thia-1-azabicyclo[4.2.0]oct-2-en-7-yl)amino)-1-(methoxyimino)-2-oxoethyl)thiazol-2-yl)amino)-1-methoxy-1,4-dioxobutan-2-yl)-1,4,7-triazonane-1,4-diyl)diacetic acid (6)

The removal of the tertbutyl groups was achieved by dissolving compound **6** (20 mg, 0.02 mmol) in thioanisole (86.4 µL, 4.0 mL/mmol), DCM (475 µL, 22 mL/mmol) and TFA (475 µL, 22 mL/mmol). The reaction was left in a freezer at minus 20 degrees for 72 hrs. During this time the reaction vessel was swirled every 24 hrs until completion. Upon completion the TFA was removed under a stream of nitrogen in the fume hood and washed three times with cold diethyl ether (~ 3.0 mL). The precipitate was isolated using centrifugation and the pellet was dried under a gentle stream of argon gas, until it was a free-flowing powder. An Off-white powder was obtained with a yield of 89% (47 mg).

Confirmed with LC-MS, m/z = 813 $(m+H)^+$. IR ($\upsilon max/cm^{-1}$) 3392, 3257, 2949, 2872, 1722, 1667, 1544, 1434, 1375, 1260, 1169, 1122, 1103. HRMS (ESI+) m/z $(m+H)^+$ calculated for $C_{31}H_{40}N_8O_{14}S_2$: 813.2283; found: 813.2344

Computational Methods

System preparation

The wild-type NDM-1 and VIM-2 single x-ray crystal structures from bacterial strains were retrieved from the protein data bank (PDB ID 5LSC & 4RL0) [4,5]. The crystal water molecules were removed, and only chain A was considered for each enzyme. Both enzymes have two Zn^{2+} metal ions in the active site trivalently coordinated by the histidine's (HIS), aspartate (ASP), and cysteine (CYS) amino acid residues. The atomic positions of the crystal-bound ligands in the active site of both enzymes were used to generate the grid box and later removed. The NDM-1 enzyme has a catalytic water molecule participating in the coordination of the two zinc metal ions. The BP 2 compound was modeled using the GaussView program and further optimized with the DFT B3LYP/6-31+G(d) level of theory.

Molecular docking of BP 2 into NDM-1 and VIM-2

Molecular interaction analysis of BP 2 compound with both NDM-1 and VIM-2 was performed by molecular docking using AutoDock Vina [6] implemented in UCSF Chimera 1.15 [7]. The grid box generated using the active site ligand position was used to dock the BP 2 compound, and the docking exhaustiveness was set to eight with an energy interval of three. The docking

results were ranked according to their scores combined with the RMSD values. The best conformation was selected based on visual inspection (using chemical knowledge) and the RMSD cut-off of <2 Å.

MD simulation

Post molecular docking, the bond orders' assignment and hydrogenation for the complexes were performed using Protein Preparation Wizard in Schrodinger Maestro (Schrödinger Release 2021-4: Maestro, Schrödinger, LLC, 2021). The ionization state of the BP 2 was determined using Epik at a suitable pH of 7.0 ± 2.0 [8]. The protonation state was determined using PROPKA embedded in Maestro [9]. The restrained energy minimization of the complexes was performed using the OPLS4 force field [10]. MD simulations were performed using Desmond [11] to assess the interactions and evaluate the binding free energy profiles of the complexes. The "System Setup" utility in Maestro was used to set up all the energy minimized systems by placing them in an orthorhombic box with a buffer distance of 10 Å. A TIP3P [12] solvation model with a 9 Å cut-off for van der Waals was used with time step, initial temperature, and pressure of the systems set to 2.0 fs, 300K, and 1.01325 bar, respectively, neutralized with a 0.15 M NaCl buffer. Furthermore, the sampling interval during the simulation was set to 50 ps, and the MD simulations were performed under the NPT ensemble for 1 μs.

Post-MD trajectory analyses

The MD trajectories were analyzed using the "simulation interaction diagram" tool in Maestro to assess the interactions between the BP 2 and the enzymes investigated in this study. The root-mean-square deviation (RMSD), root-mean-square fluctuation (RMSF), and solvent accessible surface area (SASA) were assessed to provide the systems' stability, dynamic behavior (fluctuations), and solvent accessibility metrics, respectively, during the MD simulation run. The Desmond trajectory clustering tool in Maestro was used to attain

representative structures for calculating the binding free energy. The extraction interval was 10 frames, and 2000 frames were used for clustering after MD simulations. The Prime MMGBSA tool in Maestro was used to conduct the binding free energy calculations. The VSGB solvation model [13] and OPLS4 force field [10] were set for binding free energy calculation.

Calculation of DMSO content for BP 2 preparation for the MIC:

5 mg of BP 2 dissolves in 50 % DMSO to make a 1 mL stock

256 µl of this BP 2 stock is diluted in 744 µl water to make a 1 mL working solution of 128 mg/L (12.8% DMSO)

700 µl of the working solution is diluted in 700 µl water to yield 1.4 mL of the desired concentration, 64 mg/L (6.4 % DMSO)

10 µl of the above desired concentration is added to each well of the micro titre plate with a final well volume of a 100 µl (0.64% DMSO)

Multiple dosing of animals for *in vivo* efficacy study per treatment regimen; S, M or BP 2:

Group 1 of mice received 0 dose, n=30

Group 2 of mice received 1 dose, n=24

Group 3 of mice received 2 doses, n=18

Group 4 of mice received 3 doses, n=12

Group 5 of mice received 4 doses, n=6

Figures, Tables and Spectra

A

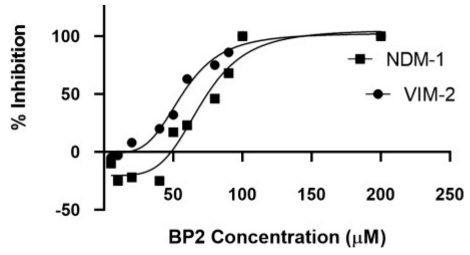

B

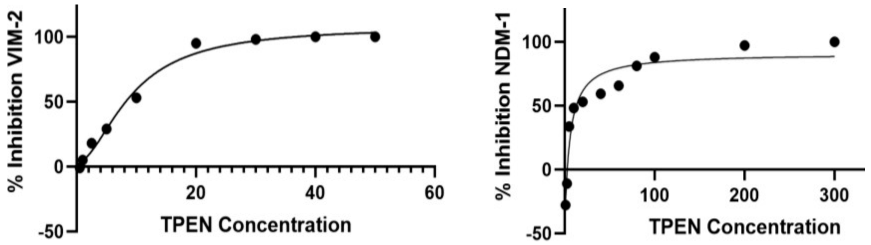

C

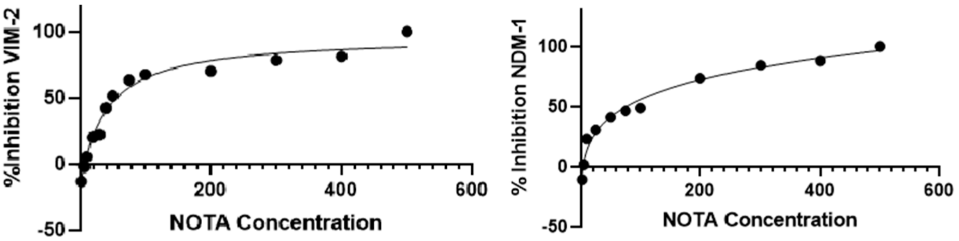

Figure S1: Half the maximal inhibitory concentration of BP 2 (A), TPEN(B) and NOTA (C) against NDM-1 and VIM-2. TPEN and NOTA were used as positive controls in the assay.

Table S1: ***Klebsiella pneumoniae*** **NDM infected mice receiving the placebo.**

Treatment Time (h)	Best Log10 Cfu/ml	Mean Log10 Cfu/ml	Std Dev	RSD (%)
2	8.1	8.7	0.8	9.7
4	11.3	11.7	0.6	5.1
6	12.6	12.6	1	8

8	12.8	13	0.7	5.3

Table S2: *Klebsiella pneumoniae* NDM infected mice receiving meropenem only treatment

Treatment Time (h)	Best Log10 Cfu/ml	Mean Log10 Cfu/ml	Std Dev	RSD (%)
2	7.2	6.8	0.4	6.5
4	8	8.3	0.5	5.5
6	8.3	8.4	0.3	3.6
8	8.3	8.2	0.2	2.1

Table S3: *Klebsiella pneumoniae* NDM infected mice receiving BP 2 and meropenem treatment

Treatment Time (h)	Best Log10 Cfu/ml	Mean Log10 Cfu/ml	Std Dev	RSD (%)
2	4.9	5.5	0.4	7.2
4	4.7	4.8	0.1	2.1
6	4	4.5	0.2	5.2
8	3.9	4	0.1	2.7

Table S4: Summary of plasma drug concentrations from the *in vivo* efficacy study

Time (Hour)	BP 2 (ng/mL)	Meropenem (ng/mL)	SD	SD
0	0	0	0	0
2	2674	7061	137	719
4	6969	27496	1606	3185
6	4427	14857	1686	1631
8	3486	8838	1821	1091

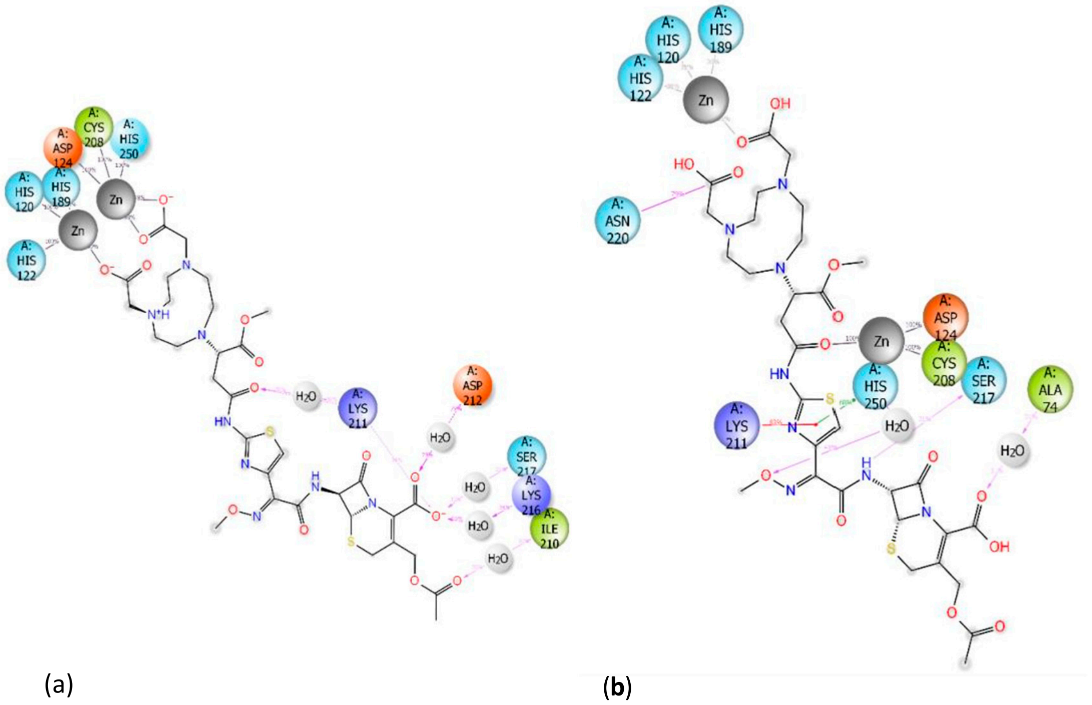

(a) (b)

Figure S1. Interaction diagrams of the investigated systems (a) NDM-1—BP 2_SR, (b) NDM-1—BP 2_SS, (c) VIM-2—BP 2_SR, and (d) VIM-2—BP 2_SS

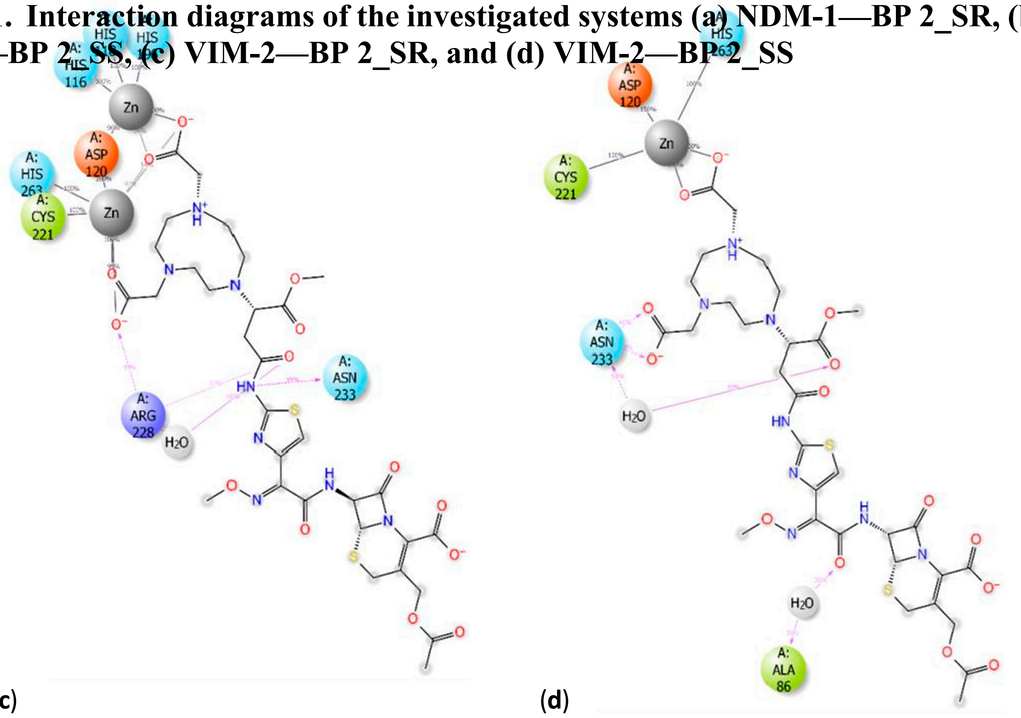

(c) (d)

Table S5: Docking scores and the binding free energies for NDM-1—BP 2 and VIM-2—BP 2 complexes

Complexes	Docking scores (kJ/mol)	ΔG$_{bind}$ (kJ/mol)
**NDM-1—BP 2_SR**	-27.20	-40.79
**NDM-1—BP 2_SS**	-25.94	-53.76
**VIM-2—BP 2_SR**	-29.71	-22.43
**VIM-2—BP 2_SS**	-26.78	-72.34

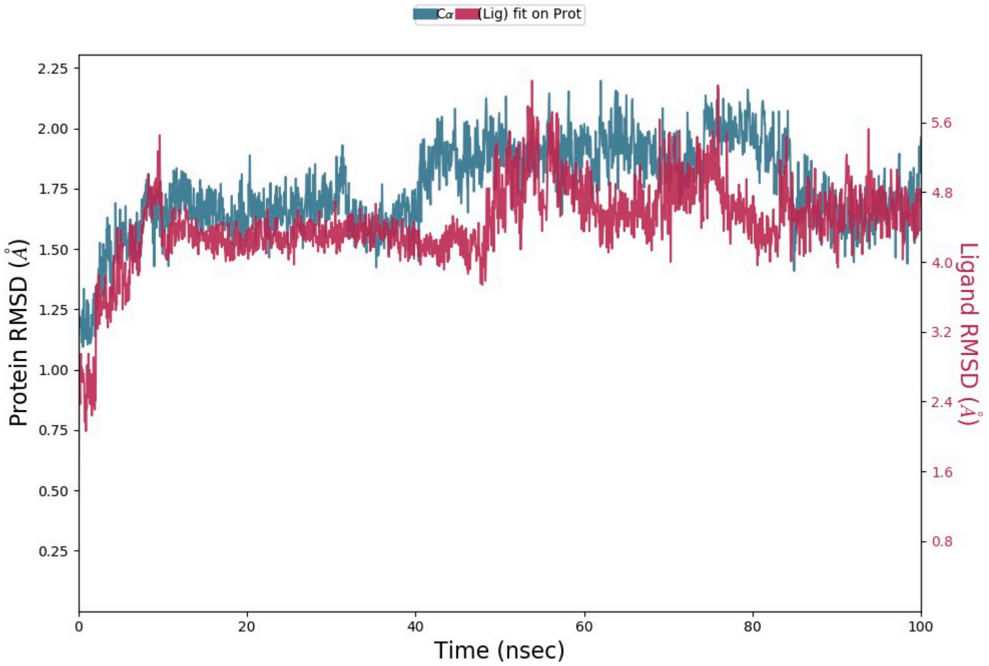

Figure S3: RMSD plot for NDM-1 Ca-backbone aligned with BP 2_SR

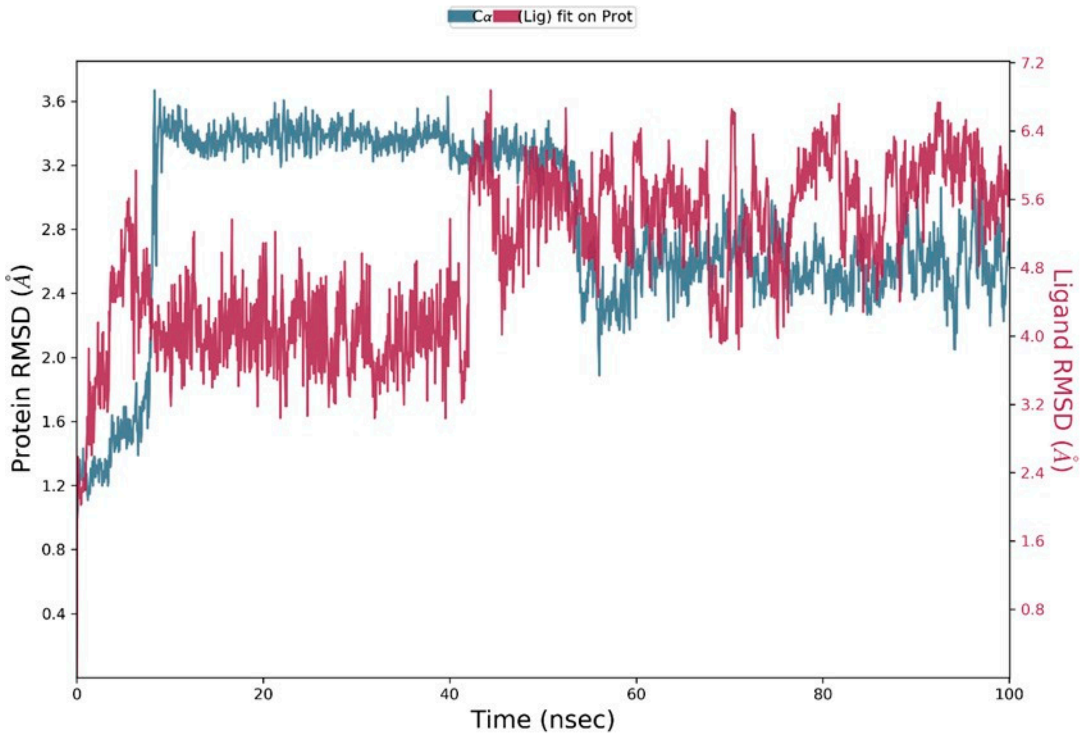

Figure S4: RMSD plot for NDM-1—BP 2_SS complex

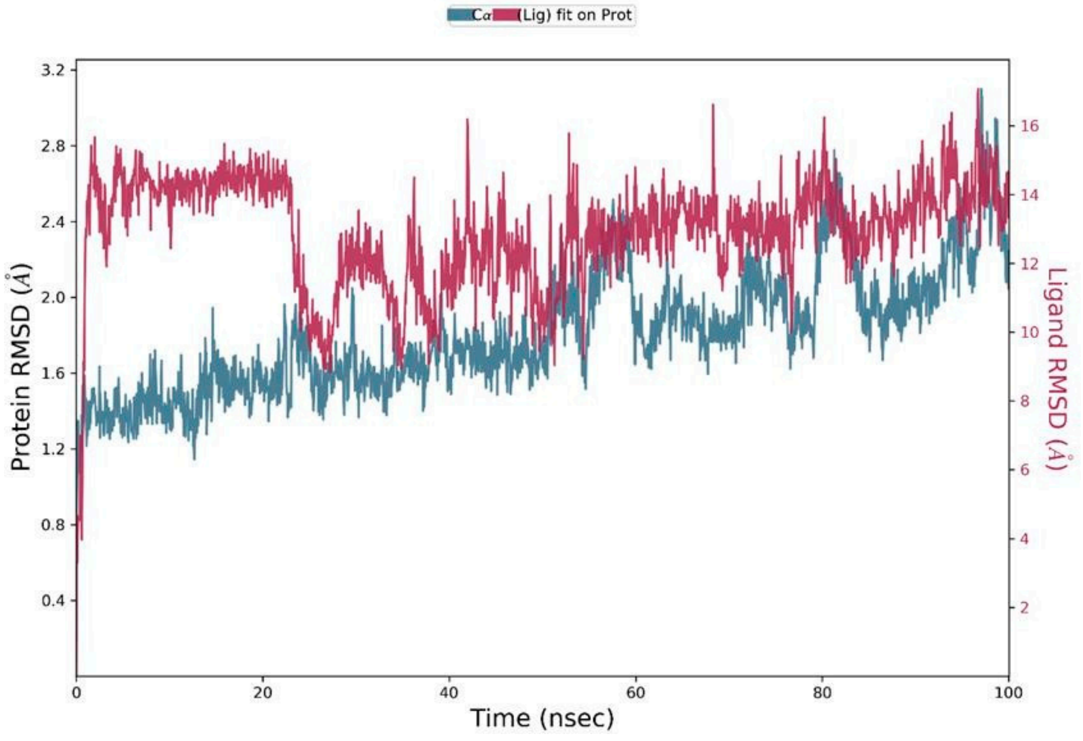

Figure S5: RMSD plot for VIM-2—BP 2_SR complex

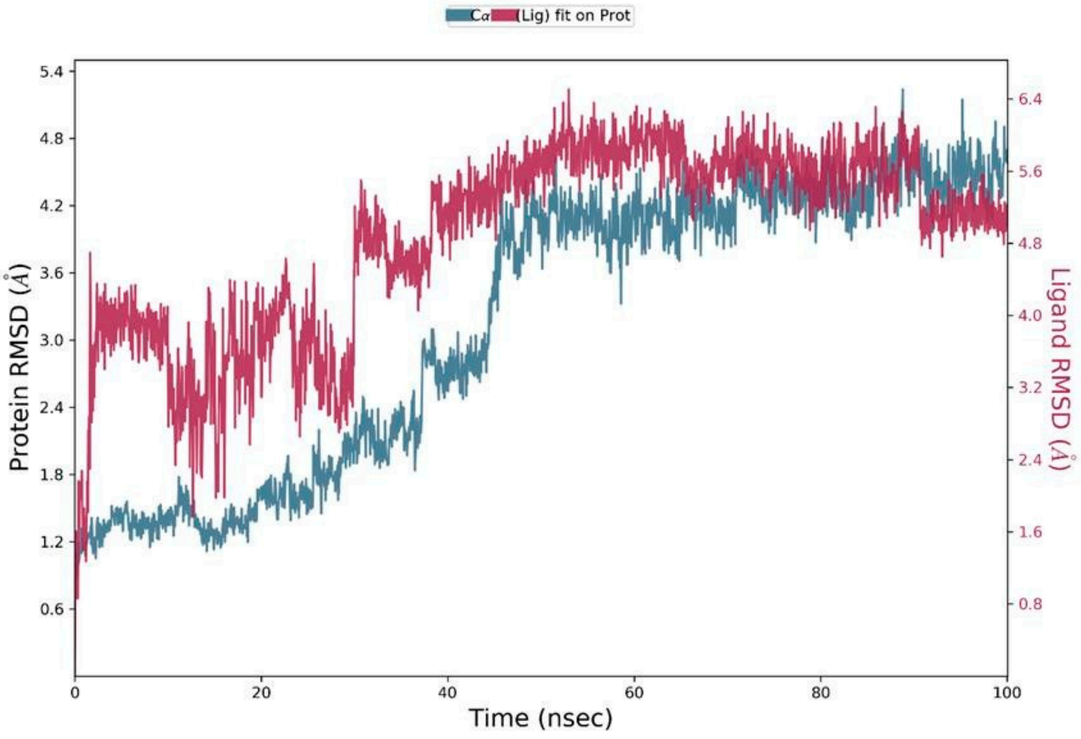

Figure S6: RMSD plot for VIM-2—BP 2_SS complex

Spectra ^{1}H NMR of cefotaxime

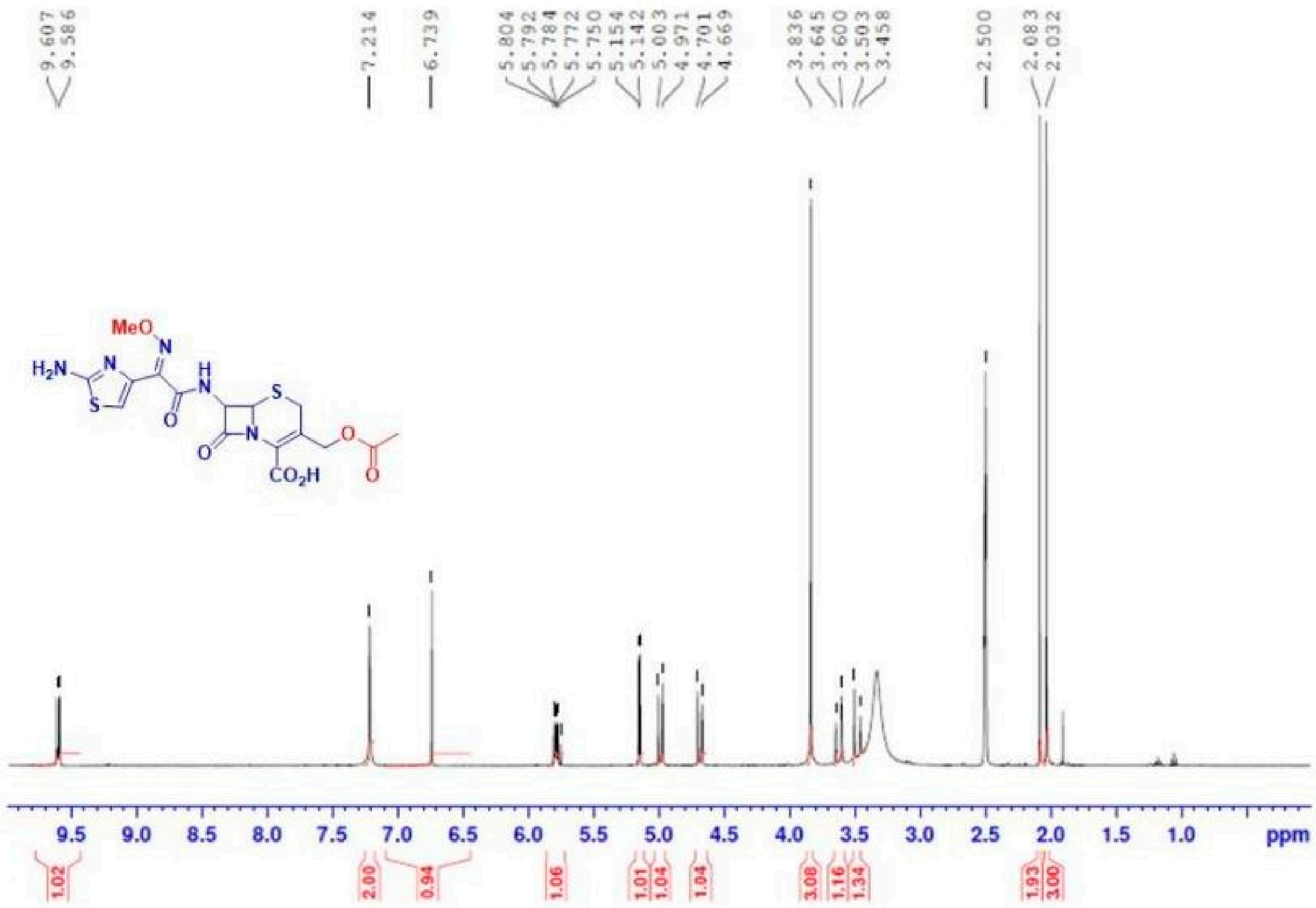

^{13}C NMR of cefotaxime

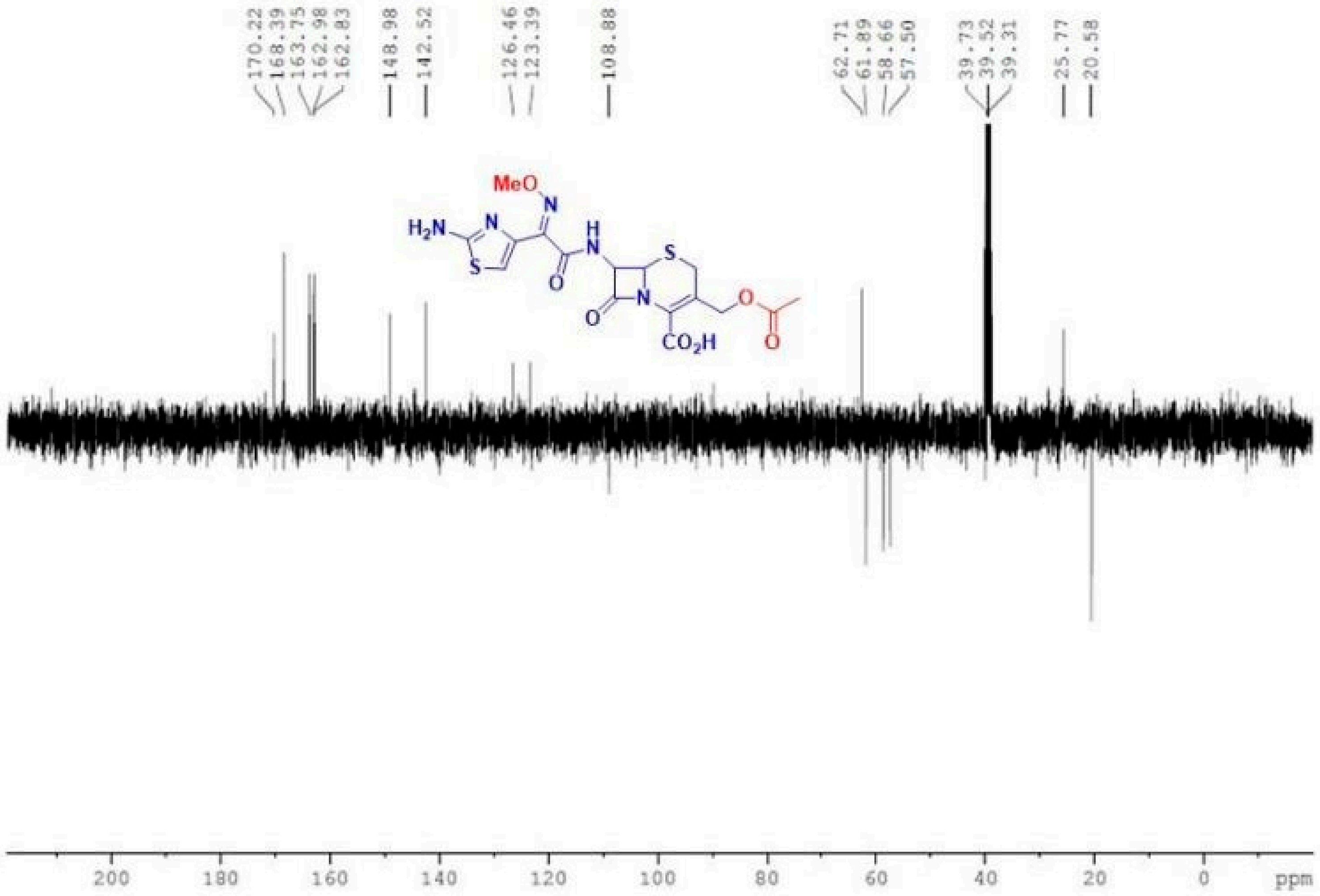

IR spectrum of **3**

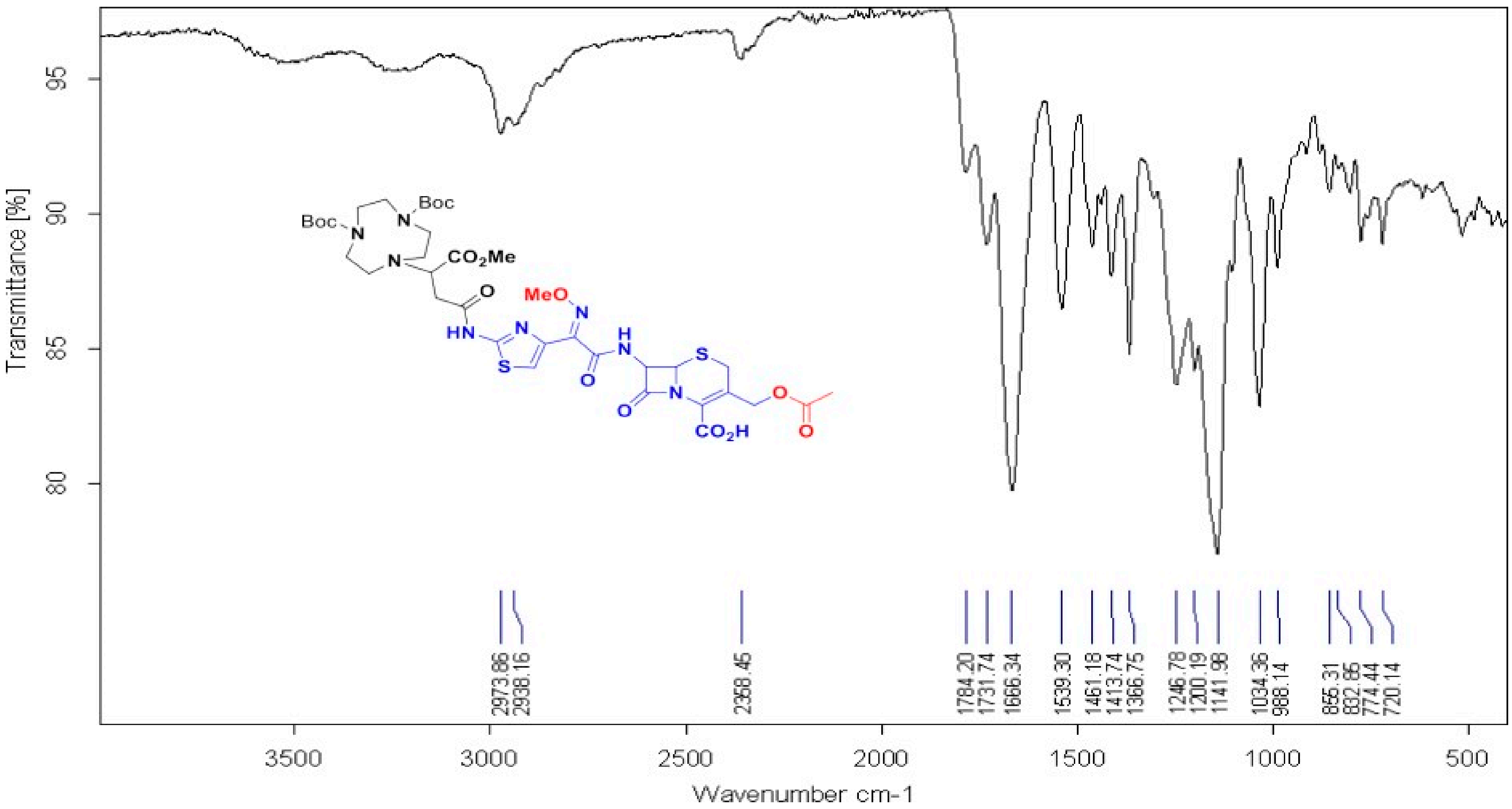

HRM Spectrum of **3**

Acquisition Parameter

Source Type	ESI	Ion Polarity	Positive	Set Nebulizer	1.5 Bar
Focus	Not active	Set Capillary	5500 V	Set Dry Heater	220 °C
Scan Begin	150 m/z	Set End Plate Offset	-500 V	Set Dry Gas	8.0 l/min
Scan End	1600 m/z	Set Collision Cell RF	400.0 Vpp	Set Divert Valve	Source

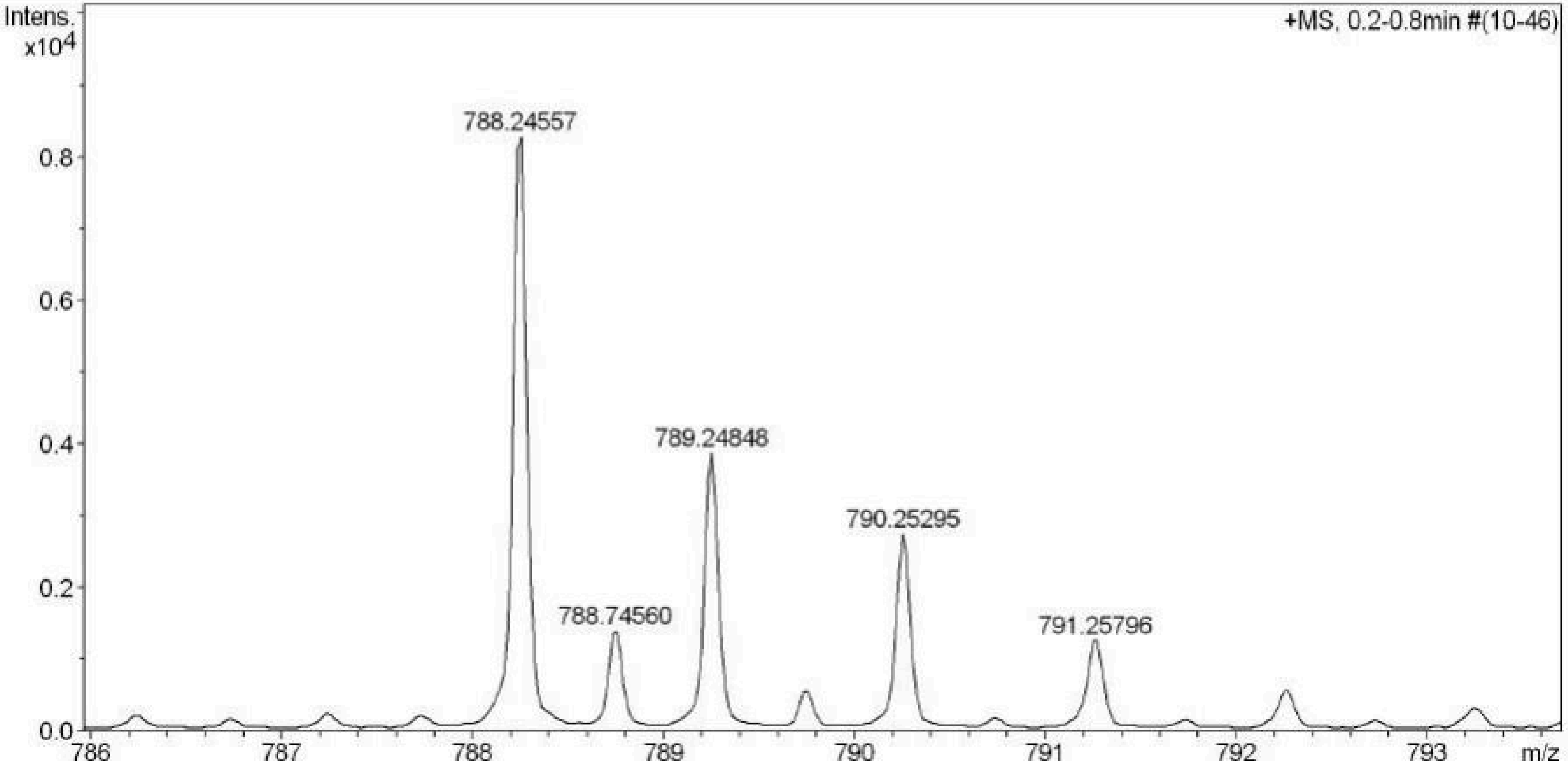

IR Spectrum of **4**

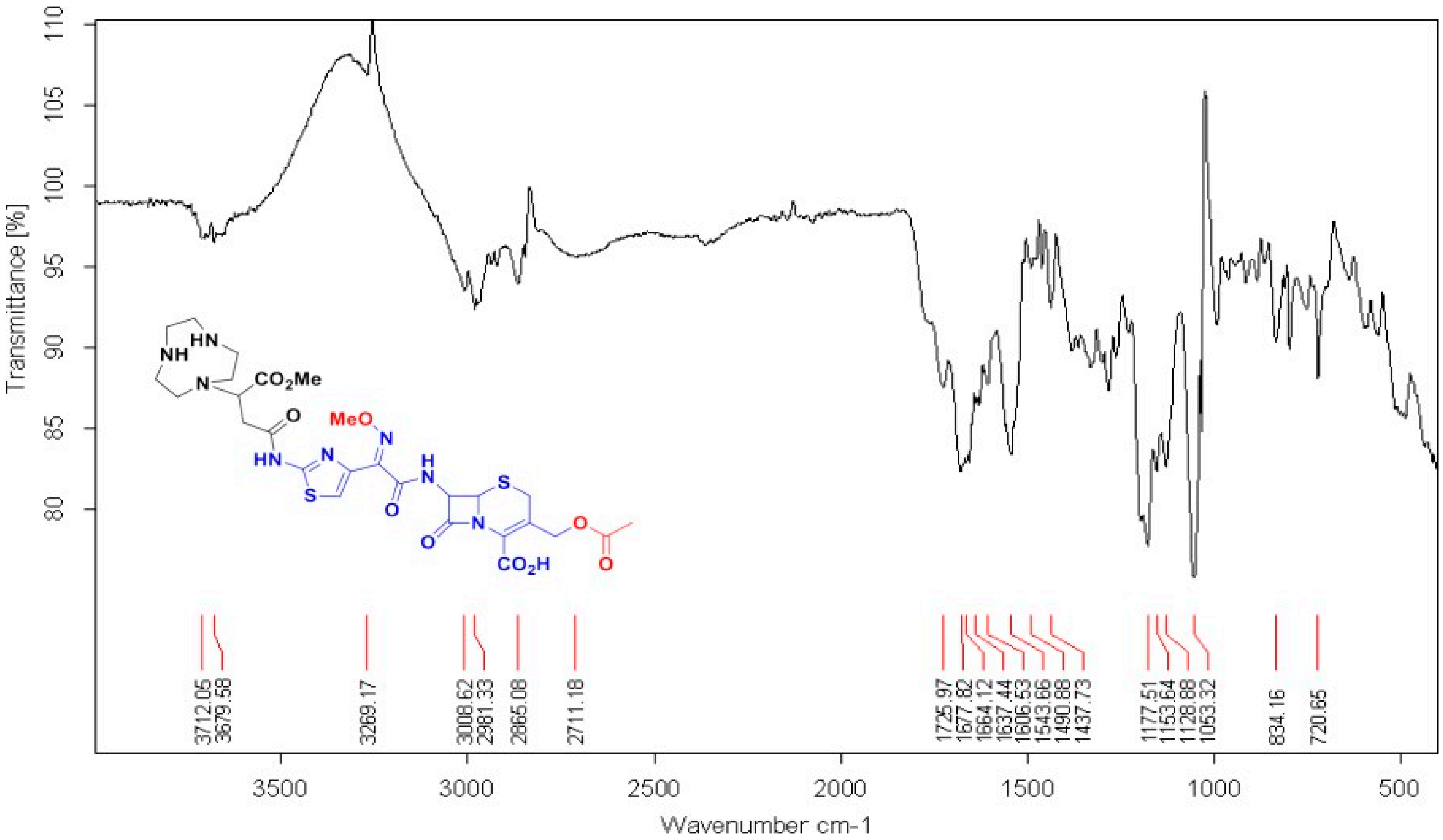

HRM Spectrum of **4**

Acquisition Parameter

Source Type	ESI	Ion Polarity	Positive	Set Nebulizer	1.5 Bar
Focus	Active	Set Capillary	5500 V	Set Dry Heater	180 °C
Scan Begin	50 m/z	Set End Plate Offset	-500 V	Set Dry Gas	8.0 l/min
Scan End	2600 m/z	Set Collision Cell RF	500.0 Vpp	Set Divert Valve	Source

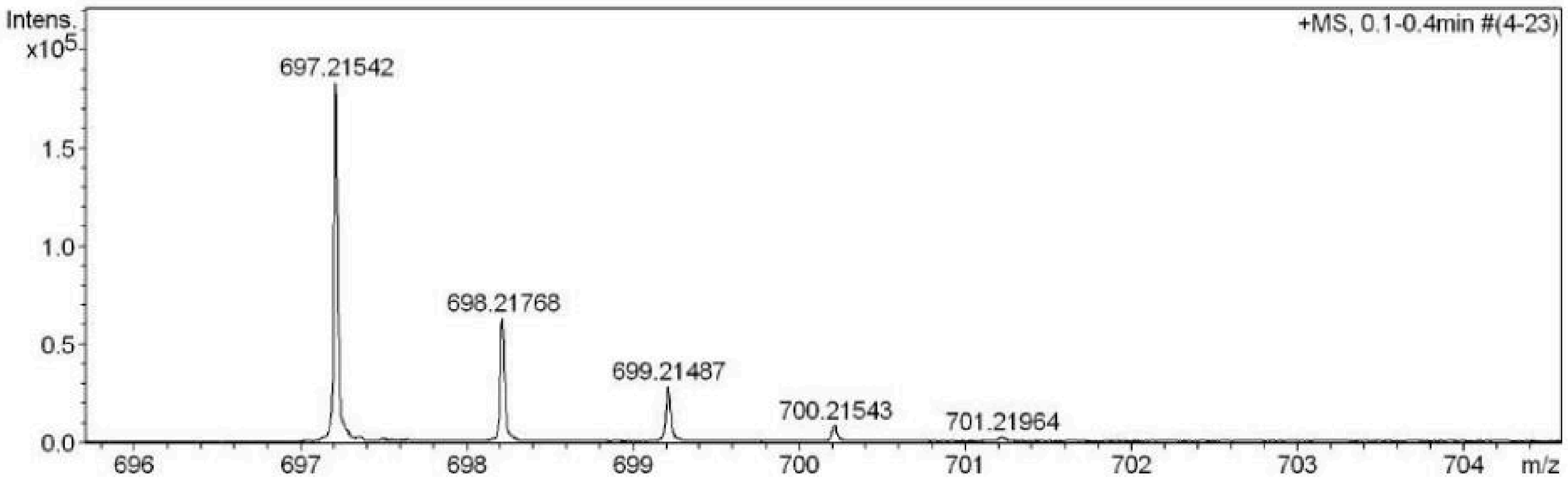

IR Spectrum of **5**

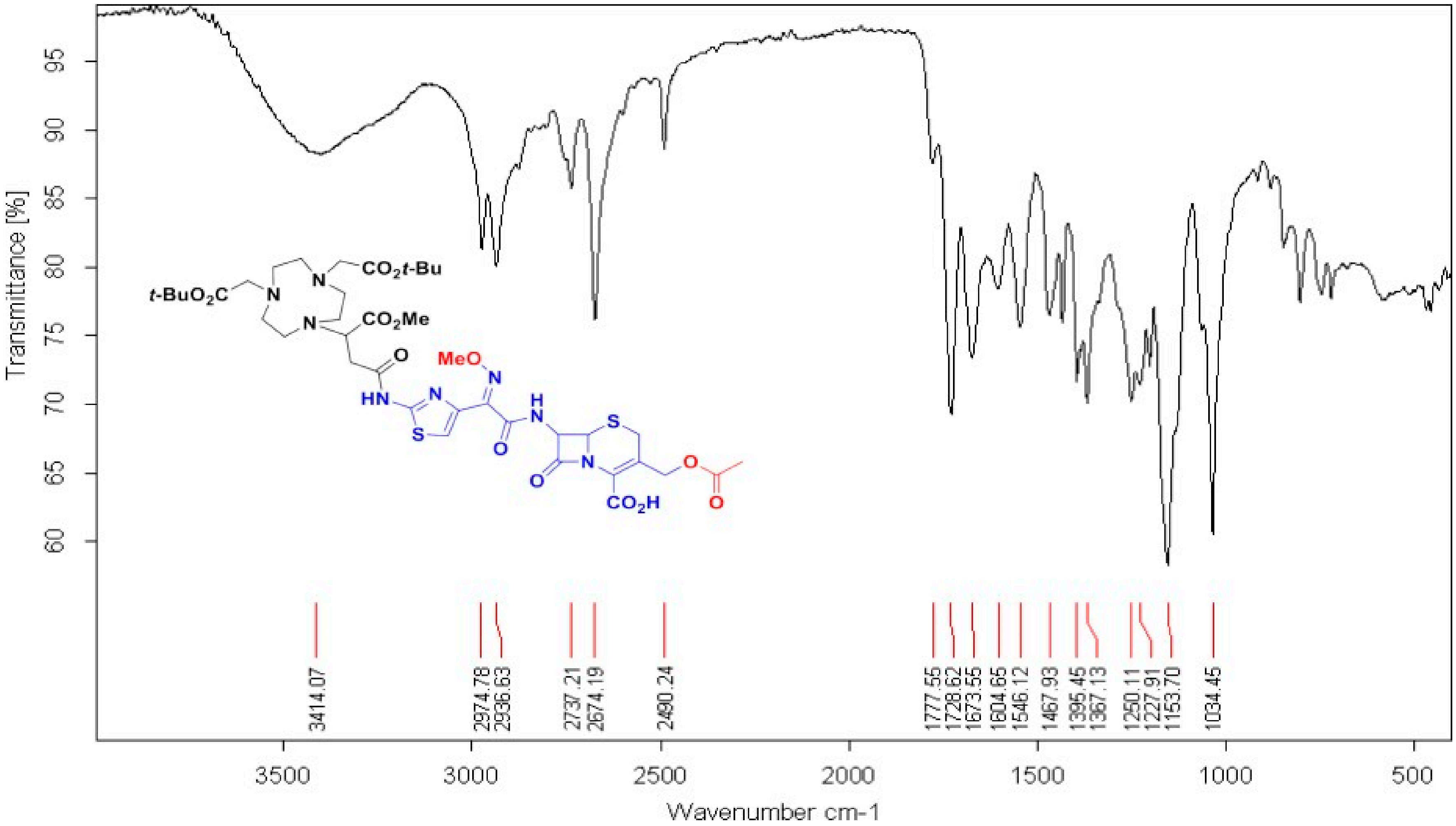

HRM Spectrum of **5**

Acquisition Parameter

Source Type	ESI	Ion Polarity	Positive	Set Nebulizer	1.5 Bar
Focus	Active	Set Capillary	5500 V	Set Dry Heater	180 °C
Scan Begin	50 m/z	Set End Plate Offset	-500 V	Set Dry Gas	8.0 l/min
Scan End	2600 m/z	Set Collision Cell RF	500.0 Vpp	Set Divert Valve	Source

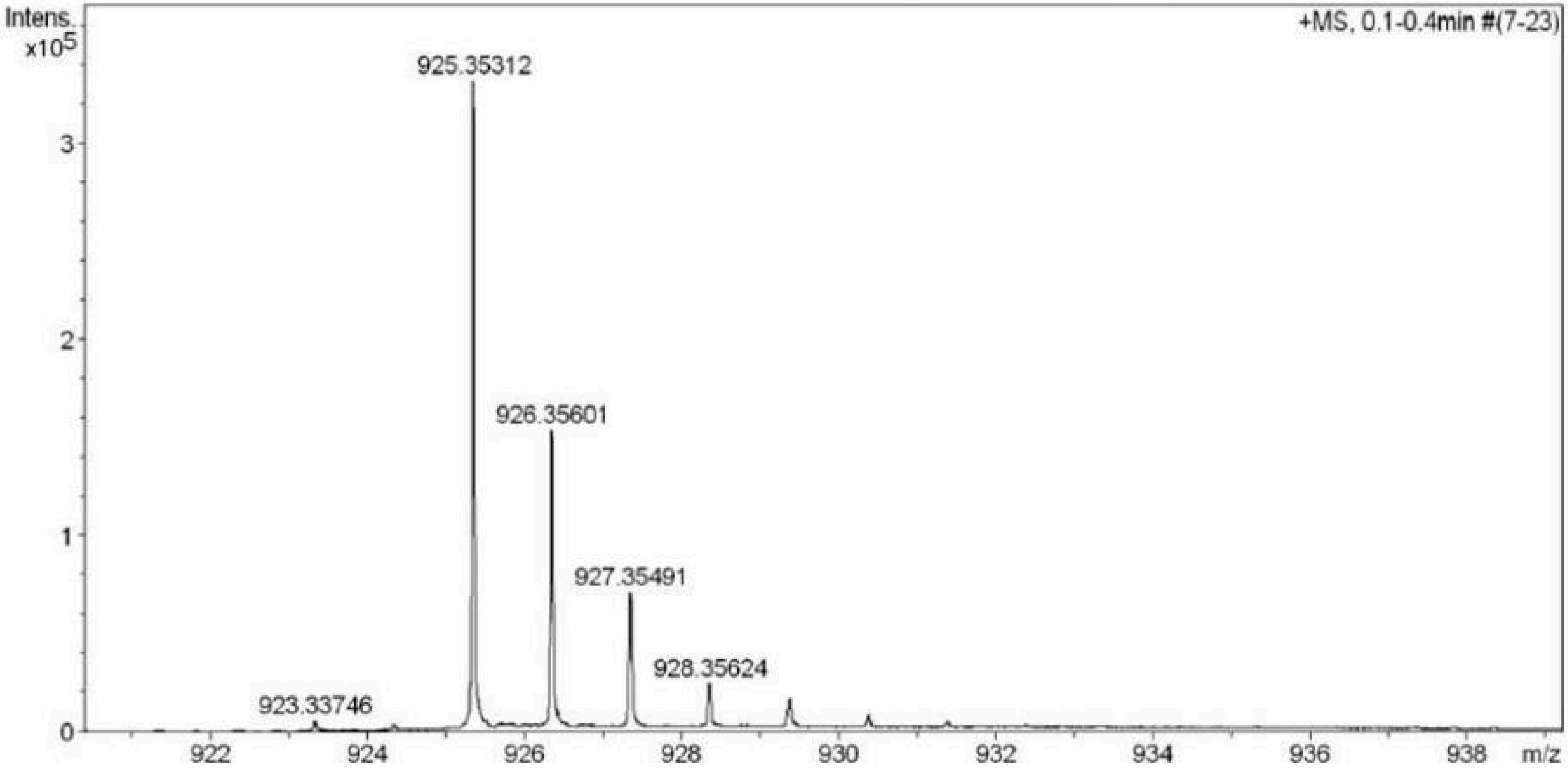

IR Spectrum of **6**

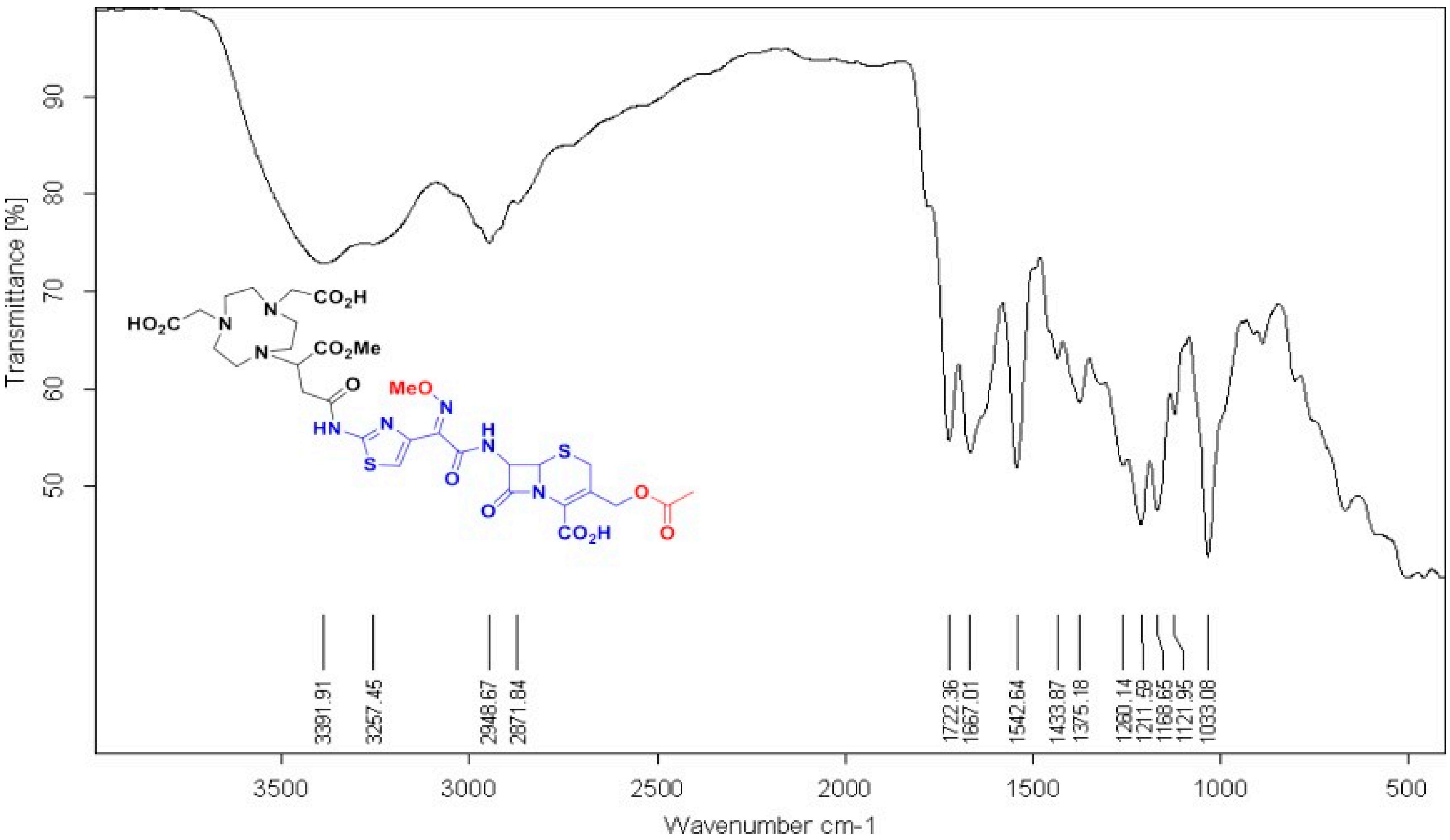

HRM Spectrum of **6**

Acquisition Parameter

Source Type	ESI	Ion Polarity	Positive	Set Nebulizer	1.5 Bar
Focus	Active	Set Capillary	5500 V	Set Dry Heater	180 °C
Scan Begin	50 m/z	Set End Plate Offset	-500 V	Set Dry Gas	8.0 l/min
Scan End	2600 m/z	Set Collision Cell RF	500.0 Vpp	Set Divert Valve	Source

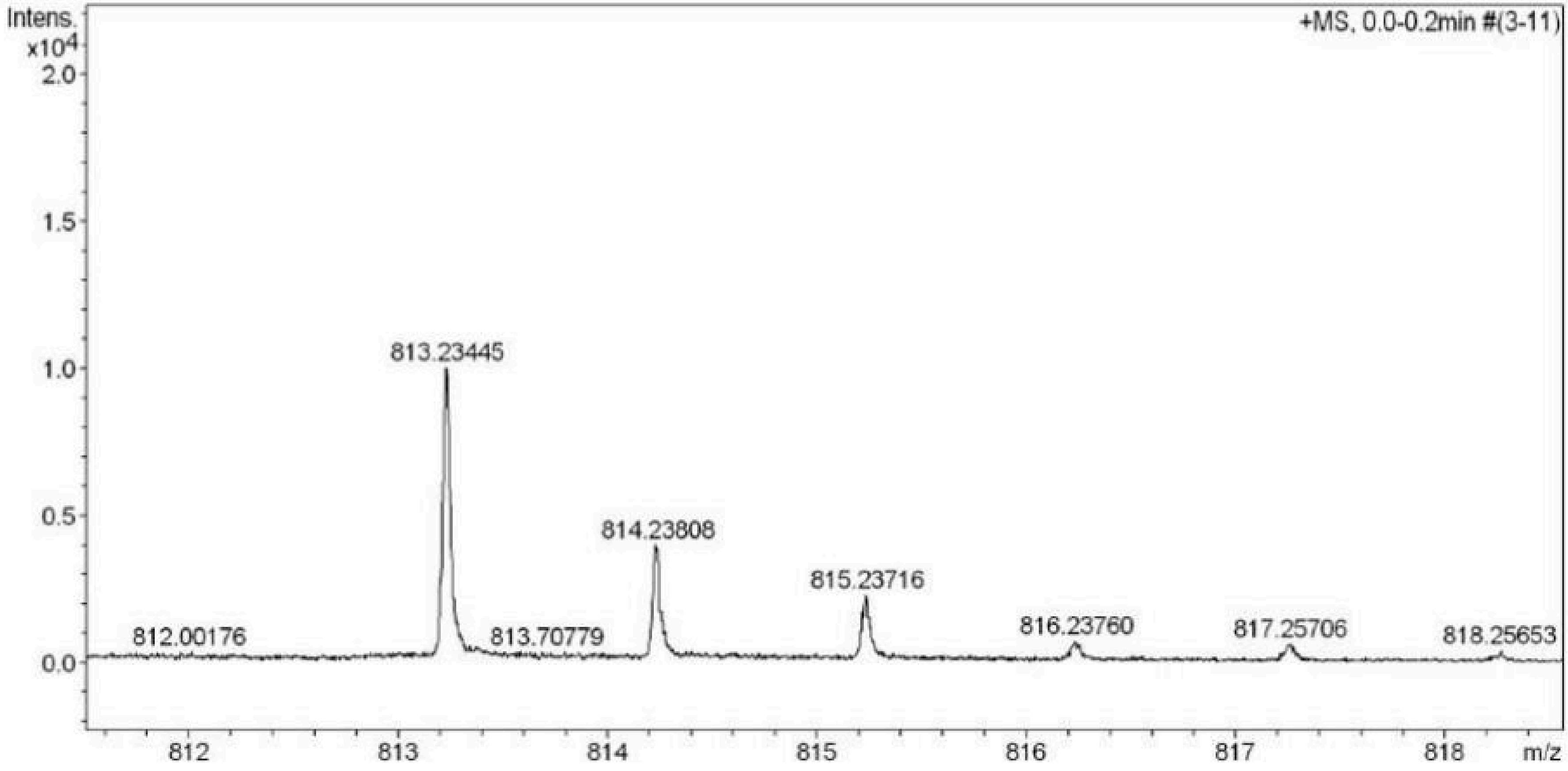

LCMS Chromatogram of **6**

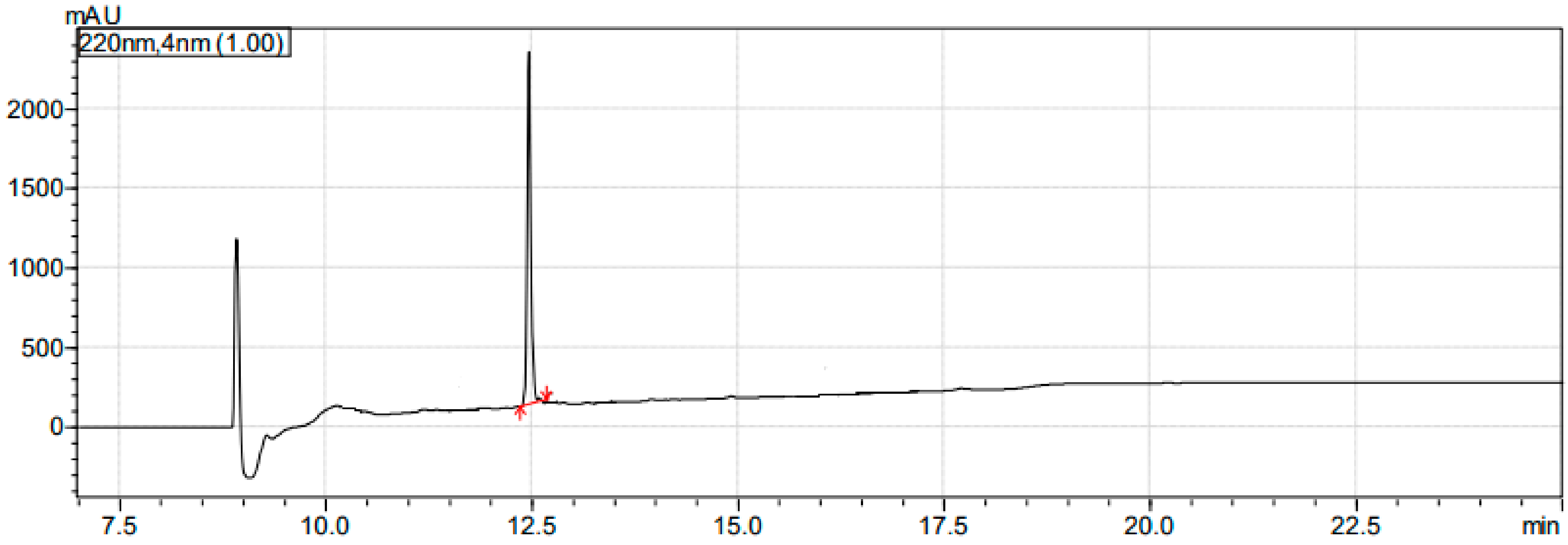

Reference

65. Hu, D.X.; Grice, P.; Ley, S.V. Rotamers or Diastereomers? An Overlooked NMR Solution. *J. Org. Chem.* **2012**, *77*, 5198–5202, https://doi.org/10.1021/jo300734r.
66. Petzoldlab. Available online: http://petzoldlab.com (accessed on 1st April 2022).
67. W., A.J. n.m.r. and chemistry; Chapman and Hall Ltd. CUP Archive: London, 1973.
68. Christopeit, T.; Yang, K.-W.; Yang, S.-K.; Leiros, H.-K.S. The structure of the metallo-β-lactamase VIM-2 in complex with a triazolylthioacetamide inhibitor. Acta Crystallogr. *Sect. F Struct. Biol. Commun.* **2016**, *72*, 813–819, https://doi.org/10.1107/s2053230x16016113.
69. Feng, H.; Ding, J.; Zhu, D.; Liu, X.; Xu, X.; Zhang, Y.; Zang, S.; Wang, D.-C.; Liu, W. Structural and mechanistic insights into NDM-1 catalyzed hydrolysis of cephalosporins. *J. Am. Chem. Soc.* **2014**, *136*, 14694–14697.
70. Trott, O.; Olson, A.J. AutoDock Vina: improving the speed and accuracy of docking with a new scoring function, efficient optimization, and multithreading. *J. Theor. Comput. Chem.* **2010**, *31*, 455–461.
71. Pettersen, E.F.; Goddard, T.D.; Huang, C.C.; Couch, G.S.; Greenblatt, D.M.; Meng, E.C.; Ferrin, T.E. UCSF Chimera—a visualization system for exploratory research and analysis. *J. Theor. Comput. Chem.* **2004**, *25*, 1605–1612.

72. Shelley, J.C.; Cholleti, A.; Frye, L.L.; Greenwood, J.R.; Timlin, M.R.; Uchimaya, M. Epik: a software program for pK a prediction and protonation state generation for drug-like molecules. *J. Comput. Aided Mol. Des.* **2007**, *21*, 681–691.
73. Li, H.; Robertson, A.D.; Jensen, J.H. Very fast empirical prediction and rationalization of protein pKa values. *Proteins*. **2005**, *61*, 704–721.
74. Lu, C.; Wu, C.; Ghoreishi, D.; Chen, W.; Wang, L.; Damm, W.; Ross, G.A.; Dahlgren, M.K.; Russell, E.; Von Bargen, C.D. OPLS4: Improving force field accuracy on challenging regimes of chemical space. *J. Chem. Theory Comput.* **2021**, *17*, 4291– 4300.
75. Release, S. 3: Desmond molecular dynamics system. DE Shaw Research, New York, NY 2017.
76. Price, D.J.; Brooks III, C.L. A modified TIP3P water potential for simulation with Ewald summation. *J. Chem. Phys*. **2004**, *121*, 10096–10103.
77. Li, J.; Abel, R.; Zhu, K.; Cao, Y.; Zhao, S.; Friesner, R.A. The VSGB 2.0 model: a next generation energy model for high resolution protein structure modeling. *Proteins*. **2011**, *79*, 2794–2812.
